# Associations Between Pregnancy-Related Predisposing Factors for Offspring Neurodevelopmental Conditions and Parental Genetic Liability to Attention-Deficit/Hyperactivity Disorder, Autism, and Schizophrenia: The Norwegian Mother, Father and Child Cohort Study (MoBa)

**DOI:** 10.1001/jamapsychiatry.2022.1728

**Published:** 2022-07-06

**Authors:** Alexandra Havdahl, Robyn E. Wootton, Beate Leppert, Lucy Riglin, Helga Ask, Martin Tesli, Ragna Bugge Askeland, Laurie J. Hannigan, Elizabeth Corfield, Anne-Siri Øyen, Ole A. Andreassen, Kate Tilling, George Davey Smith, Anita Thapar, Ted Reichborn-Kjennerud, Evie Stergiakouli

**Affiliations:** 1 Nic Waals Institute, Lovisenberg Diaconal Hospital, Oslo, Norway; 2 Department of Mental Disorders, Norwegian Institute of Public Health, Oslo, Norway; 3 PROMENTA, Department of Psychology, University of Oslo, Oslo, Norway; 4 MRC (Medical Research Council) Integrative Epidemiology Unit, University of Bristol, Bristol, United Kingdom; 5 Population Health Sciences, Bristol Medical School, University of Bristol, Bristol, United Kingdom; 6 Division of Psychological Medicine and Clinical Neurosciences, MRC Centre for Neuropsychiatric Genetics and Genomics, Cardiff University, Cardiff, United Kingdom; 7 Wolfson Centre for Young People’s Mental Health, Cardiff University, Cardiff, United Kingdom; 8 NORMENT, Institute of Clinical Medicine, University of Oslo, Oslo, Norway; 9 Division of Mental Health and Addiction, Oslo University Hospital, Oslo, Norway; 10 Institute of Clinical Medicine, University of Oslo, Oslo, Norway

## Abstract

**Importance:**

Several maternal exposures during pregnancy are considered predisposing factors for offspring neurodevelopmental conditions. However, many of these exposures may be noncausal and biased by maternal genetic liability.

**Objective:**

To assess whether pregnancy-related predisposing factors for offspring neurodevelopmental conditions are associated with maternal genetic liability for attention-deficit/hyperactivity disorder (ADHD), autism, and schizophrenia and to compare associations for maternal genetic liability with those for paternal genetic liability, which could indicate that paternal exposures are not suitable negative controls for maternal exposures.

**Design, Setting, and Participants:**

The Norwegian Mother, Father and Child Cohort Study (MoBa) is a population-based pregnancy cohort that recruited parents from June 1999 to December 2008. Polygenic scores (PGS) for ADHD, autism, and schizophrenia were derived in mothers and fathers. The associations between maternal PGS and 37 pregnancy-related measures were estimated, and these results were compared with those from paternal PGS predicting paternal measures during the mother’s pregnancy. Analysis took place between March 2021 and March 2022.

**Exposures:**

PGS for ADHD, autism, and schizophrenia, calculated (using discovery effect size estimates and threshold of *P* < .05) from the largest available genome-wide association studies.

**Main Outcomes and Measures:**

Self-reported pregnancy-related measures capturing lifestyle behaviors, metabolism, infectious and autoimmune diseases, other physical health conditions, and medication use.

**Results:**

Data were available for up to 14 539 mothers (mean [SD] age, 30.00 [4.45] years) and 14 897 fathers (mean [SD] age, 32.46 [5.13] years) of European ancestry. Modest but robust associations were observed between specific pregnancy-related measures and maternal PGS, including ADHD PGS with asthma (odds ratio [OR], 1.15 [95% CI, 1.06-1.25]), smoking (OR, 1.26 [95% CI, 1.19-1.33]), prepregnancy body mass index (β, 0.25 [95% CI, 0.18-0.31]), pregnancy weight gain (β, 0.20 [95% CI, 0.10-0.30]), taking folate (OR, 0.92 [95% CI, 0.88-0.96]), and not taking supplements (OR, 1.09 [95% CI, 1.04-1.14]). Schizophrenia PGS was associated with coffee consumption (OR, 1.09 [95% CI, 1.05-1.12]), smoking (OR, 1.12 [95% CI, 1.06-1.19]), prepregnancy body mass index (β, −0.18 [95% CI, −0.25 to −0.11]), and pregnancy weight gain (β, 0.17 [95% CI, 0.07-0.27]). All 3 PGSs associated with symptoms of depression/anxiety (ADHD: OR, 1.15 [95% CI, 1.09-1.22]; autism: OR, 1.13 [95% CI, 1.06-1.19]; schizophrenia: OR, 1.13 [95% CI, 1.07-1.20]). Associations were largely consistent for maternal and paternal PGS, except ADHD PGS and smoking (fathers: OR, 1.13 [95% CI, 1.09-1.17]).

**Conclusions and Relevance:**

In this study, genetic liability to neurodevelopmental conditions that is passed from mothers to children was associated with several pregnancy-related factors and may therefore confound associations between these pregnancy-related factors and offspring neurodevelopment that have previously been thought to be causal. It is crucial that future study designs account for genetic confounding to obtain valid causal inferences so that accurate advice can be given to pregnant individuals.

## Key Points

**Question:**

Does maternal genetic liability for attention-deficit/hyperactivity disorder, autism, and schizophrenia predict exposure to pregnancy factors hypothesized to be causal for neurodevelopmental conditions in offspring?

**Findings:**

In this cohort study of 14 539 mothers and 14 897 fathers, associations between polygenic scores for attention-deficit/hyperactivity disorder, autism, and schizophrenia and 37 pregnancy-related predisposing factors were assessed. Higher genetic liability in mothers was found to be modestly but robustly associated with likelihood of experiencing several of the pregnancy-related factors associated with offspring neurodevelopmental conditions.

**Meaning:**

Observed associations between some pregnancy-related factors and offspring neurodevelopmental conditions are likely subject to genetic confounding and may not be causal.

## Teaser

This cohort study explores whether pregnancy-related predisposing factors for offspring neurodevelopmental conditions are associated with maternal genetic liability for attention-deficit/hyperactivity disorder (ADHD), autism, and schizophrenia

## Introduction

Attention-deficit/hyperactivity disorder (ADHD) and autism spectrum disorder (hereafter, autism) are common neurodevelopmental conditions.^1^ Schizophrenia, although with later onset, shares many of the same features, and is hypothesized to have a neurodevelopmental origin. ADHD, autism, and schizophrenia are all highly heritable,^2^ although their etiology is complex and likely to include a combination of genetic, environmental, and stochastic factors.^1^ As development of these conditions is thought to begin early, prenatal factors have been investigated widely as potential predisposing factors, often through observational studies.^3,4,5,6,7,8^ However, it is unclear whether the observed associations are causal. Observed associations may reflect confounding by unknown or unmeasured shared familial factors that influence maternal exposure and offspring neurodevelopmental outcomes.^9,10^ Genetic confounding, when the same genetic factors are independently associated with both the exposure and outcome, has consistently been shown to explain the association between smoking during pregnancy and risk for ADHD in children using different causally informative designs.^11,12,13,14,15^ Determining if exposure to prenatal factors is causal is important because misleading evidence about the causes of neurodevelopmental conditions can result in unnecessary worry for pregnant individuals and hinder attention to more appropriate intervention targets.

Genome-wide association studies (GWAS) have revealed ADHD, autism, and schizophrenia to be highly polygenic.^16,17,18^ Individual common genetic liability can be expressed by a composite measure, called a polygenic score (PGS), summarizing the association of all the risk-increasing genetic variants identified in the GWAS.^19^ By using PGS for genetic liability to neurodevelopmental conditions to predict pregnancy exposures, we can test whether shared genetics transmitted from mother to offspring can partially explain associations in observational studies.^20^

Comparison of maternal and paternal PGS associations with the same pregnancy-related factors is informative given that paternal exposures are commonly used as negative controls to strengthen causal inference about intrauterine effects of maternal exposures.^21^ Under the assumption that maternal and paternal associations between exposures and neurodevelopmental outcomes in the child are similarly influenced by shared genetics between parent and offspring (and other confounding familial factors, eg, socioeconomic status), similar magnitudes of parental factor–offspring outcome associations would suggest that the maternal factor does not have a causal intrauterine effect. However, different magnitudes of association between maternal vs paternal PGS with exposures would suggest that the use of paternal exposures as a negative control may not be appropriate. For example, some negative control studies have found stronger associations between prenatal substance use (smoking, alcohol, and caffeine) and offspring ADHD for mothers compared with fathers,^15,22^ despite broader triangulation of evidence suggesting no causal effects.^12,13,15^ During pregnancy, the substance use of the pregnant individual is a more severe phenotype than that of their nonpregnant partner, owing to the strong pressure on pregnant individuals to quit. Therefore, a higher genetic liability for substance use might be required for pregnant individuals to continue to smoke compared with their partners.

In the current study, we tested for the association between maternal and paternal PGS for ADHD, autism, and schizophrenia with maternal pregnancy-related exposures and co-occurring paternal exposures in the Norwegian Mother, Father and Child Cohort Study (MoBa).^23^ The large sample size of mothers and fathers with genetic data allows us to include rare pregnancy-related factors such as vitamin B_12_ insufficiency and use of depression medication. We also compare PGS associations with maternal exposures before and during pregnancy.

## Methods

### Sample

We used data from MoBa, a population-based pregnancy cohort study conducted by the Norwegian Institute of Public Health.^23^ Participants were recruited from all over Norway from June 1999 to December 2008. Individuals were classified as mothers or fathers based on whether they returned the questionnaire for mothers or fathers. The genetic data quality control identified all of the mothers and fathers in the sample used in this article as chromosomally female and male, respectively. Mothers consented in writing to participate in 41% of the pregnancies. The cohort includes approximately 114 500 children, 95 200 mothers, and 75 200 fathers. The current study is based on version 12 of the quality-assured data files released for research in January 2019. The establishment of MoBa and initial data collection was based on a license from the Norwegian Data Protection Agency and approval from the Regional Committees for Medical and Health Research Ethics. The MoBa cohort is now based on regulations related to the Norwegian Health Registry Act. The current study was approved by the Regional Committees for Medical and Health Research Ethics (2016/1702). Blood samples were collected from both parents during pregnancy and from children (umbilical cord) at birth.^24^ A range of pregnancy-related factors have been captured through questionnaires and the Medical Birth Registry of Norway, a national health registry containing information about all births in Norway. Genotyping and quality control are described in eAppendix 1 in the Supplement and previously.^25^ Quality-controlled genotype data were available for 14 804 mothers and 15 198 fathers of European ancestry.

### Polygenic Scores

Of the neurodevelopmental conditions, only ADHD and autism had available GWAS summary statistics of large sample sizes (>10 000 cases). Schizophrenia was additionally included because of its commonly shared features of atypical attention, activity and impulse regulation, social communication and behavioral flexibility, hypothesized neurodevelopmental origin, and shared observational associations with prenatal exposures and to allow comparison with results from a study in the Avon Longitudinal Study of Parents and Children (ALSPAC) cohort.^26^

Maternal and paternal PGS for ADHD, autism, and schizophrenia were calculated using PRSice version 2.0^27^ as the weighted sum of single-nucleotide variants (only common sequence variants, ie, single-nucleotide polymorphisms) associated with ADHD, autism and schizophrenia in the discovery GWAS.^20^ We excluded single-nucleotide variants in approximate linkage disequilibrium (*r^2^* < 0.25 within a 500-kb sliding window) and in the major histocompatibility complex region owing to complex linkage disequilibrium structure. PGSs were adjusted for genotyping batch and the top 10 principal components to adjust for population stratification. Standardized residual scores were used in all analyses. Risk alleles were identified in GWAS for ADHD (individuals with ADHD, n = 20 183; controls, n = 35 191),^16^ autism (individuals with autism, n = 18 381; controls, n = 27 969)^17^ and schizophrenia (individuals with schizophrenia, n = 36 989; controls, n = 113 075).^18^ Our primary single-nucleotide variant inclusion *P* value threshold was less than .05, selected for comparability with relevant previous studies of these PGS.^20,28^ eAppendix 2 in the Supplement shows the number of single-nucleotide variants included in each PGS and histograms and correlations between PGS.

### Pregnancy-Related Factors

Pregnancy-related factors were chosen after a literature review of early life exposures that have been reported to be associated with neurodevelopmental conditions (Table 1; eAppendix 3 in the Supplement). Broadly, these exposures related to maternal lifestyle and health behaviors, metabolism, immune system, other physical health conditions, and medication use. We excluded pregnancy-related factors with fewer than 100 cases based on our power calculation (eAppendix 3 in the Supplement). Where the same measures were reported by the fathers (during the mother’s pregnancy), we included these in the father’s analysis (eAppendix 3 in the Supplement).

**Table 1.** Sample Overview and Comparison of Maternal Pregnancy-Related Factors in the Full and Genotyped Norwegian Mother, Father and Child Cohort Study Cohort

Characteristic	Full sample, No. (%)		Genotyped sample, No. (%)		*P* value^a^
	No	Yes	No	Yes	
Female child	48 453 (51.2)	46 180 (48.8)	7418 (51.0)	7113 (49)	.70
Male child	46180 (48.8)	48453 (51.2)	7113 (49.0)	7418 (51.0)	.70
Behavior and lifestyle					
Cigarette smoking	78 208 (89.7)	9008 (10.3)	13 286 (91.6)	1219 (8.4)	8.38 × 10^−17^
Alcohol consumption	59 826 (68.6)	27 390 (31.4)	9921 (68.4)	4584 (31.6)	.58
Binge drinking	74 841 (86.1)	12 077 (13.9)	12 365 (85.3)	2138 (14.7)	.001
Coffee consumption	48 942 (56.3)	37 976 (43.7)	8302 (57.2)	6201 (42.8)	.01
Binge coffee drinking	85 242 (98.1)	1676 (1.9)	14 307 (98.6)	196 (1.4)	3.78 × 10^−8^
No supplements taken	69 842 (80.4)	17 076 (19.6)	11 878 (81.9)	2625 (18.1)	3.00 × 10^−7^
Folate supplement before pregnancy	49 868 (57.9)	36 211 (42.1)	7877 (54.4)	6616 (45.6)	1.06 × 10^−21^
Folate supplement during pregnancy	17 863 (20.8)	68 216 (79.2)	2334 (16.1)	12 159 (83.9)	1.23 × 10^−51^
Metabolic conditions					
Type 2 diabetes (including gestational diabetes)	93 543 (98.7)	1265 (1.3)	14 365 (98.8)	174 (1.2)	.13
High blood pressure (including preeclampsia)	83 138 (87.7)	11 670 (12.3)	12 549 (86.3)	1990 (13.7)	4.17 × 10^−8^
Hyperthyroidism/hypothyroidism	84 461 (98.1)	1618 (1.9)	14 252 (98.3)	241 (1.7)	.04
Infectious and autoimmune diseases					
Upper respiratory tract infections	75 768 (86.9)	11 448 (13.1)	12 666 (87.3)	1839 (12.7)	.08
Lower respiratory tract infections	84 625 (97.0)	2591 (3)	14 116 (97.3)	389 (2.7)	.03
Urinary tract infection	78 843 (90.4)	8373 (9.6)	13 107 (90.4)	1398 (9.6)	.88
Fever	73 832 (84.7)	13 384 (15.3)	12 271 (84.6)	2234 (15.4)	.85
Asthma	83 360 (95.9)	3558 (4.1)	13 924 (96.0)	579 (4)	.52
Psoriasis	84 600 (98.3)	1479 (1.7)	14 233 (98.2)	260 (1.8)	.46
Type 1 diabetes	94 432 (99.6)	376 (0.4)	14 484 (99.6)	55 (0.4)	.76
Other autoimmune disease	84 827 (98.5)	1252 (1.5)	14 276 (98.5)	217 (1.5)	.66
Other physical health conditions					
Vaginal bleeding	94 640 (99.8)	168 (0.2)	14 513 (99.8)	26 (0.2)	>.99
Vitamin B_12_ insufficiency	84 346 (98.0)	1733 (2)	14 229 (98.2)	264 (1.8)	.08
Anemia/low hemoglobin in early pregnancy	83 601 (97.1)	2478 (2.9)	14 130 (97.5)	363 (2.5)	.003
Indication for medicine use					
Depression/anxiety	77 251 (88.9)	9667 (11.1)	13 180 (90.9)	1323 (9.1)	5.42 × 10^−17^
Depression medication	85 868 (98.8)	1050 (1.2)	14 361 (99.0)	142 (1)	.006
Depression or anxiety medication	85 627 (98.5)	1291 (1.5)	14 328 (98.8)	175 (1.2)	.003
Pain	24 432 (28.1)	62 487 (71.9)	3946 (27.2)	10 557 (72.8)	.008
Migraine	89 935 (94.9)	4873 (5.1)	13 753 (94.6)	786 (5.4)	.12
Headache	67 347 (78.2)	18 733 (21.8)	11 320 (78.1)	3173 (21.9)	.68
Epilepsy	85 816 (99.7)	263 (0.3)	14 456 (99.7)	37 (0.3)	.26
Pain medication	81 093 (93.3)	5825 (6.7)	13 588 (93.6)	915 (6.3)	.04
Fever medication	84 579 (98.3)	1500 (1.7)	14 243 (98.3)	250 (1.7)	.89
Pain or fever medication	79 763 (91.8)	7155 (8.2)	13 369 (92.2)	1134 (7.8)	.049
Paracetamol use	42 927 (49.4)	43 991 (50.6)	7031 (48.5)	7472 (51.5)	.02
Ibuprofen use	80 433 (92.5)	6485 (7.5)	13 362 (92.1)	1141 (7.9)	.04

^a^ *P* values from χ^2^ test comparing the genotyped sample with the nongenotyped sample. Multiple testing correction threshold was *P* < .002. Unless otherwise specified, the variables were measured during pregnancy.

### Statistical Analysis

We restricted the sample to 1 observation per mother, keeping the firstborn child in MoBa. The multiple testing corrected threshold for significance was determined to be *P* < .002 for all analyses (eAppendix 4 in the Supplement). Analysis took place between March 2021 and March 2022.

#### PGS Validation

We assessed whether the ADHD PGS predicted ADHD behaviors in the MoBa parents (eAppendix 7 in the Supplement). No direct phenotypic measure of autism or schizophrenia behaviors was available for the parents. However, autism and schizophrenia PGSs have both been validated in previous samples.^29,30,31^

#### Primary Analyses

Associations of maternal ADHD, autism, and schizophrenia PGS with pregnancy-related factors were assessed using linear regression for continuous measures and logistic regression for binary outcomes in Stata version 15.1 (StataCorp). Effect estimates are presented per 1-SD increase in PGS. Analyses were repeated for paternal PGS on available paternal pregnancy-related factors.

#### Secondary Analyses

To assess consistency, where possible, we estimated the association between maternal neurodevelopmental PGS and relevant exposures before pregnancy and at specific trimesters of the pregnancy.

#### Sensitivity Analyses

PGS constructed using *P* value thresholds .0005, .005, .05, .10, and .50 were derived for sensitivity analyses as they provide different balance between levels of variance explained and inclusion of pleiotropic variants. We also conducted 2 analyses to investigate the potential effect of missing data. First, inverse probability weighting on missing maternal genetic data was applied to account for sampling bias in the genotyped data set (eAppendix 5 in the Supplement). Second, we used multiple imputation (n = 100) with chained equations to impute missing data in the PGS and pregnancy-related factors (eAppendix 6 in the Supplement).

## Results

### Sample Overview

Data were available for up to 14 539 mothers and 14 897 fathers. To account for differences between the genotyped and nongenotyped samples (Table 1) and missing data, additional analyses were conducted (eAppendices 5 and 6 in the Supplement).

### Maternal PGS and Pregnancy-Related Exposures

Effect sizes for all associations of maternal PGS with maternal pregnancy-related exposures are shown in Table 2. Maternal ADHD PGS was associated with younger age at childbirth (of first included MoBa child), higher odds of smoking during pregnancy, higher body mass index (BMI) before pregnancy, and higher pregnancy weight gain. Higher maternal ADHD PGS was associated with lower odds of taking supplements (including folate) during pregnancy. Additionally, mothers with higher ADHD PGS were more likely to have asthma and depression/anxiety symptoms. There was weak evidence of association with higher odds of migraine and pain during pregnancy.

**Table 2.** Association of Maternal PGS for ADHD, Autism, and Schizophrenia With Pregnancy-Related Factors

Characteristic	No.	ADHD PGS		Autism PGS		Schizophrenia PGS	
		OR (95% CI)	*P* value	OR (95% CI)	*P* value	OR (95% CI)	*P* value
Behavior and lifestyle							
Maternal age^a^	14 532	−0.21 (−0.28 to −0.14)	6.18 × 10^−9^	0.08 (0.01 to 0.15)	.04	0.05 (−0.02 to 0.12)	.18
Cigarette smoking	14 505	1.26 (1.19 to 1.33)	2.2 × 10^−14^	0.99 (0.94 to 1.05)	.78	1.12 (1.06 to 1.19)	1.10 × 10^−4^
Alcohol consumption	14 505	0.96 (0.92 to 0.99)	.01	1.04 (1.01 to 1.08)	.02	1.03 (1.00 to 1.07)	.09
Binge drinking	14 503	0.98 (0.94 to 1.02)	.36	1.01 (0.96 to 1.06)	.71	1.05 (1.00 to 1.10)	.04
Coffee consumption	14 503	0.98 (0.95 to 1.02)	.29	1.03 (1.00 to 1.07)	.04	1.09 (1.05 to 1.12)	8.92 × 10^−7^
Binge coffee drinking	14 503	1.20 (1.05 to 1.38)	.01	1.00 (0.87 to 1.16)	.95	1.15 (1.00 to 1.33)	.047
No supplements taken	14 503	1.09 (1.04 to 1.14)	7.04 × 10^−5^	1.01 (0.97 to 1.05)	.59	0.94 (0.90 to 0.98)	.002
Folate supplement before pregnancy	14 493	0.96 (0.93 to 0.99)	.01	1.02 (0.99 to 1.06)	.20	1.00 (0.97 to 1.03)	.98
Folate supplement during pregnancy	14 493	0.92 (0.88 to 0.96)	3.23 × 10^−4^	0.98 (0.94 to 1.03)	.44	1.02 (0.98 to 1.07)	.36
Metabolic conditions							
Body mass index before pregnancy^a^	14 166	0.25 (0.18 to 0.31)	7.88 × 10^−13^	0.07 (−0.00 to 0.13)	.05	−0.18 (−0.25 to −0.11)	2.26 × 10^−7^
Weight gain^a^	12 268	0.20 (0.10 to 0.30)	9.63 × 10^−5^	0.01 (−0.09 to 0.11)	.88	0.17 (0.07 to 0.27)	.001
Type 2 diabetes (including gestational diabetes)	14 539	0.92 (0.80 to 1.07)	.29	1.05 (0.91 to 1.22)	.52	0.97 (0.83 to 1.12)	.66
High blood pressure (including preeclampsia)	14 539	1.00 (0.96 to 1.05)	.92	0.99 (0.94 to 1.04)	.69	1.00 (0.96 to 1.05)	.93
Hyperthyroidism/hypothyroidism	14 493	1.12 (0.99 to 1.27)	.08	0.99 (0.88 to 1.13)	.94	1.02 (0.90 to 1.16)	.78
Infectious and autoimmune diseases							
Upper respiratory tract infections	14 505	1.04 (0.99 to 1.09)	.13	1.00 (0.96 to 1.05)	.85	1.01 (0.96 to 1.06)	.80
Lower respiratory tract infections	14 505	1.02 (0.93 to 1.13)	.64	1.04 (0.94 to 1.15)	.40	1.01 (0.91 to 1.12)	.86
Urinary tract infection	14 505	1.06 (1.01 to 1.12)	.03	1.08 (1.02 to 1.14)	.005	1.06 (1.00 to 1.12)	.053
Fever	14 505	1.03 (0.98 to 1.07)	.28	0.99 (0.95 to 1.04)	.68	1.03 (0.98 to 1.07)	.26
Asthma	14 503	1.15 (1.06 to 1.25)	8.59 × 10^−4^	1.07 (0.99 to 1.16)	.10	0.95 (0.88 to 1.04)	.26
Psoriasis	14 493	0.98 (0.87 to 1.11)	.79	1.02 (0.91 to 1.16)	.71	0.89 (0.79 to 1.01)	.06
Type 1 diabetes	14 539	0.92 (0.71 to 1.20)	.53	0.97 (0.74 to 1.26)	.80	1.01 (0.77 to 1.31)	.97
Other autoimmune disease	14 493	0.94 (0.82 to 1.07)	.35	1.04 (0.91 to 1.19)	.54	0.94 (0.82 to 1.07)	.35
Other physical health conditions							
Vaginal bleeding	14 539	1.25 (0.85 to 1.83)	.26	0.84 (0.57 to 1.24)	.38	1.18 (0.80 to 1.73)	.41
Vitamin B_12_ insufficiency	14 493	1.03 (0.91 to 1.16)	.68	0.92 (0.81 to 1.04)	.17	1.03 (0.92 to 1.17)	.59
Anemia/low hemoglobin in early pregnancy	14 493	0.91 (0.82 to 1.01)	.08	0.98 (0.89 to 1.09)	.77	1.04 (0.94 to 1.16)	.42
Indication for medicine use							
Lifetime depression	14 075	1.12 (1.08 to 1.17)	3.73 × 10^−9^	1.12 (1.08 to 1.16)	9.70 × 10^−9^	1.16 (1.11 to 1.20)	3.11 × 10^−13^
Depression/anxiety symptoms	14 503	1.15 (1.09 to 1.22)	5.48 × 10^−7^	1.13 (1.06 to 1.19)	3.62 × 10^−5^	1.13 (1.07 to 1.20)	1.71 × 10^−5^
Depression medication	14 503	1.17 (0.99 to 1.38)	.06	1.03 (0.87 to 1.21)	.723	1.43 (1.21 to 1.69)	2.76 × 10^−5^
Depression or anxiety medication	14 503	1.11 (0.96 to 1.29)	.17	1.04 (0.89 to 1.20)	.63	1.37 (1.18 to 1.59)	3.72 × 10^−5^
Pain	14 503	1.05 (1.02 to 1.09)	.004	1.01 (0.97 to 1.05)	.58	1.02 (0.99 to 1.06)	.19
Migraine	14 539	1.12 (1.04 to 1.20)	.002	1.10 (1.02 to 1.18)	.009	0.98 (0.92 to 1.06)	.68
Headache	14 493	1.04 (1.00 to 1.08)	.07	0.98 (0.94 to 1.02)	.27	1.01 (0.97 to 1.05)	.60
Epilepsy	14 493	0.82 (0.59 to 1.13)	.22	0.82 (0.60 to 1.14)	.24	0.94 (0.68 to 1.30)	.71
Pain medication	14 503	1.07 (1.00 to 1.15)	.04	1.04 (0.97 to 1.11)	.29	1.05 (0.98 to 1.12)	.16
Paracetamol use	14 503	1.03 (1.00 to 1.07)	.04	1.02 (0.98 to 1.05)	.31	0.99 (0.95 to 1.02)	.41
Ibuprofen use	14 503	0.99 (0.93 to 1.05)	.81	1.06 (1.00 to 1.12)	.07	0.95 (0.89 to 1.01)	.08
Fever medication	14 493	1.01 (0.89 to 1.14)	.88	1.01 (0.89 to 1.14)	.88	0.93 (0.82 to 1.06)	.29
Pain or fever medication	14 503	1.06 (0.99 to 1.12)	.08	1.02 (0.96 to 1.09)	.45	1.02 (0.96 to 1.08)	.53

Abbreviations: ADHD, attention-deficit/hyperactivity disorder; OR, odds ratio; PGS, polygenic scores.

^a^ Effect estimates for maternal age at birth, body mass index prepregnancy, and weight gain during pregnancy are shown as β per 1-SD increase in PGS. Multiple testing corrected *P *value threshold was  <.002. Measures occurred during pregnancy unless otherwise specified.

Maternal autism PGS was associated with higher odds of experiencing depression/anxiety symptoms (and weak evidence for migraine and urinary tract infection) during pregnancy. There was little evidence for other associations of autism PGS with maternal health or lifestyle during pregnancy.

Maternal schizophrenia PGS was associated with higher odds of coffee consumption and cigarette smoking during pregnancy, lower prepregnancy BMI, and higher pregnancy weight gain. Schizophrenia PGS was associated with higher odds of depression/anxiety symptoms during pregnancy and of taking medication for depression/anxiety. There was only weak evidence of association between schizophrenia PGS and higher odds of taking supplements during pregnancy.

### Comparison of Maternal and Paternal PGS Associations

Sixteen of the pregnancy-related exposures were also measured in fathers during the mother’s pregnancy. We compared the magnitude of the maternal exposure PGS association with the magnitude of the paternal exposure PGS association (Figure and eAppendix 8 in the Supplement). Nonoverlapping confidence intervals were observed only for 2 associations: (1) maternal ADHD PGS was associated with higher odds of maternal smoking in pregnancy compared with paternal ADHD PGS predicting odds of father smoking and (2) maternal schizophrenia PGS was associated with higher maternal coffee consumption during pregnancy, while there was no association in fathers.

**Figure.**
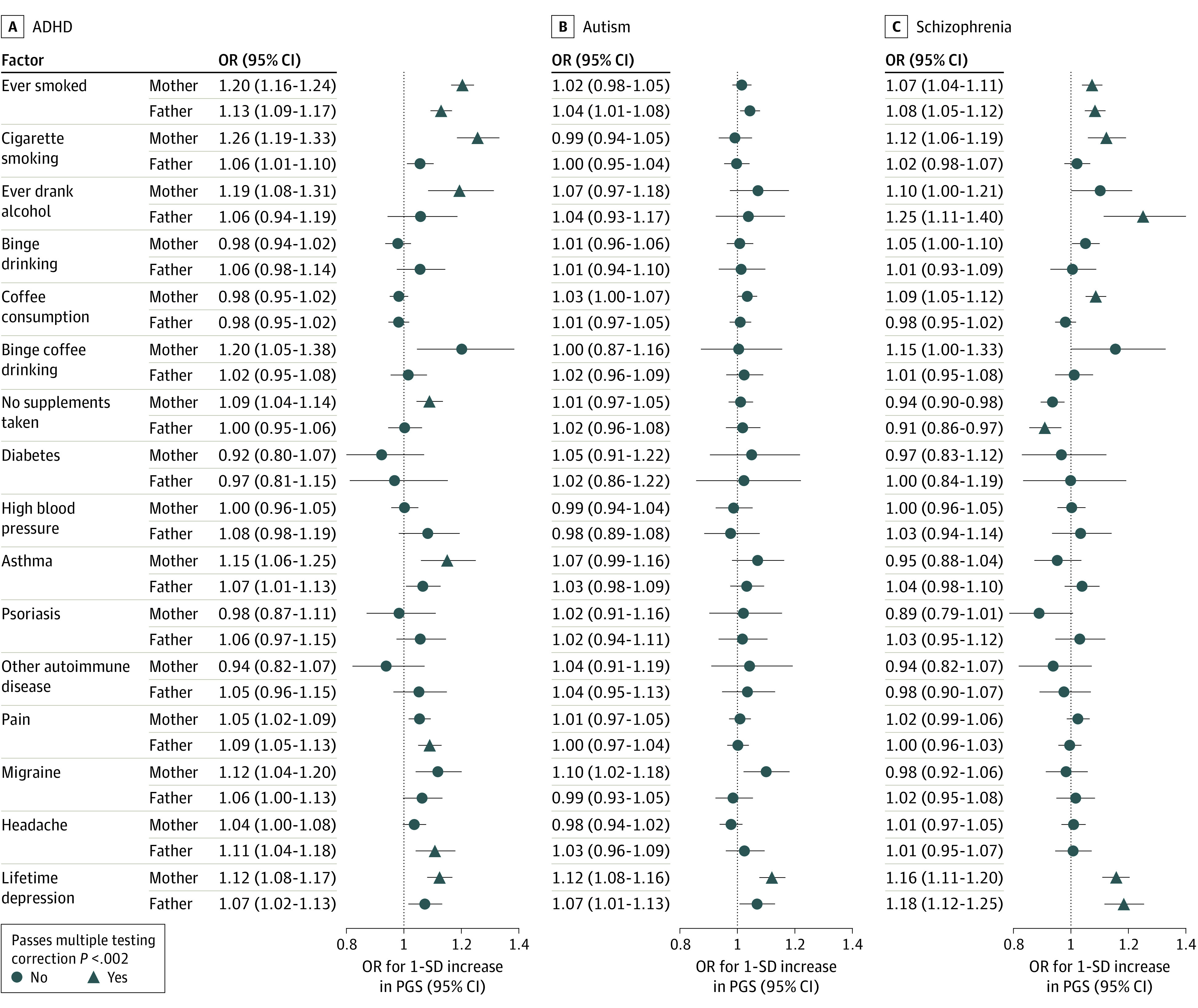
Comparison of Maternal and Paternal Polygenic Score (PGS) With Pregnancy-Related Factors ADHD indicates attention-deficit/hyperactivity disorder; OR, odds ratio.

### Secondary Analyses: Exposures Before and at Different Stages of Pregnancy

The associations of ADHD PGS with smoking, migraine, and depression before pregnancy were consistent with the associations during pregnancy (Table 3). However, mothers with higher ADHD PGS were more likely to have ever drank alcohol, whereas there was little evidence for an association with alcohol consumption during pregnancy. The associations of schizophrenia PGS with smoking and depression during pregnancy were also seen for ever smoking and lifetime depression. Autism PGS was associated with lifetime depression and depression/anxiety symptoms during pregnancy. Stratifying by trimester, differences were found for pain, with evidence that mothers with high ADHD PGS were more likely to experience pain only during the first trimester.

**Table 3.** Associations of Maternal PGS for ADHD, Autism, and Schizophrenia With Exposures by Timing of Exposure^a^

Characteristic	No.	ADHD PGS		Autism PGS		Schizophrenia PGS	
		OR (95% CI)	*P* value	OR (95% CI)	*P* value	OR (95% CI)	*P* value
**Cigarette smoking**							
Ever	14 252	1.20 (1.16-1.24)	4.00 × 10^−28^	1.02 (0.98-1.05)	.35	1.07 (1.04-1.11)	2.61 × 10^−5^
During pregnancy	14 505	1.26 (1.19-1.33)	2.29 × 10^−14^	0.99 (0.94-1.05)	.78	1.12 (1.06-1.19)	1.10 × 10^−4^
Trimester 1	12 984	1.26 (1.19-1.34)	2.43 × 10^−13^	0.99 (0.93-1.06)	.86	1.11 (1.04-1.18)	.001
Trimester 2	13 768	1.33 (1.24-1.43)	6.36 × 10^−16^	1.01 (0.94-1.08)	.76	1.09 (1.02-1.17)	.01
Trimester 3	12 700	1.28 (1.19-1.38)	7.36 × 10^−11^	1.00 (0.93-1.08)	.95	1.03 (0.96-1.11)	.43
**Alcohol consumption**							
Ever	14 182	1.19 (1.08-1.31)	2.93 × 10^−4^	1.07 (0.97-1.18)	.15	1.10 (1.00-1.21)	.048
During pregnancy	14 505	0.96 (0.92-0.99)	.01	1.04 (1.01-1.08)	.02	1.03 (1.00-1.07)	.09
Trimester 1	14 495	0.95 (0.92-0.99)	.006	1.05 (1.02-1.09)	.004	1.03 (1.00-1.07)	.07
Trimester 2	13 881	0.99 (0.93-1.05)	.67	1.07 (1.01-1.14)	.02	1.00 (0.94-1.06)	>.99
Trimester 3	13 105	0.96 (0.91-1.01)	.09	1.06 (1.00-1.11)	.04	1.02 (0.96-1.07)	.58
**High blood pressure**							
Before pregnancy	13 664	1.02 (0.97-1.07)	.48	1.06 (1.01-1.12)	.02	1.02 (0.97-1.08)	.39
During pregnancy (including preeclampsia)	14 539	1.00 (0.96-1.05)	.92	0.99 (0.94-1.04)	.69	1.00 (0.96-1.05)	.93
Trimester 1	14 493	1.01 (0.85-1.20)	.92	1.07 (0.90-1.26)	.47	1.02 (0.86-1.22)	.80
Trimester 2	13 498	1.06 (0.98-1.14)	.17	0.96 (0.88-1.04)	.27	0.98 (0.91-1.06)	.68
Trimester 3	13 105	0.99 (0.94-1.04)	.68	1.00 (0.95-1.05)	.93	1.02 (0.97-1.08)	.44
**Vaginal bleeding**							
During pregnancy	14 539	1.25 (0.85-1.83)	.26	0.84 (0.57-1.24)	.38	1.18 (0.80-1.73)	.41
Trimester 1	14 539	1.06 (0.97-1.16)	.21	1.00 (0.92-1.10)	.94	1.00 (0.92-1.10)	.94
Trimester 2	14 539	1.05 (0.91-1.21)	.51	1.10 (0.95-1.26)	.19	1.06 (0.92-1.23)	.40
Trimester 3	14 539	0.93 (0.77-1.11)	.43	1.04 (0.86-1.24)	.71	0.96 (0.80-1.16)	.70
**Urinary tract infections**							
Before pregnancy	14 493	1.01 (0.98-1.05)	.50	1.01 (0.97-1.05)	.61	1.02 (0.98-1.06)	.30
During pregnancy	14 505	1.06 (1.01-1.12)	.02	1.08 (1.02-1.14)	.005	1.06 (1.00-1.12)	.053
Trimester 1	14 493	1.04 (0.97-1.11)	.29	1.08 (1.01-1.15)	.03	1.03 (0.96-1.10)	.39
Trimester 2	13 881	1.09 (1.01-1.18)	.03	1.12 (1.04-1.21)	.004	1.09 (1.01-1.18)	.03
Trimester 3	13 881	1.05 (0.88-1.24)	.61	1.07 (0.90-1.27)	.44	1.15 (0.96-1.37)	.12
**Fever**							
During pregnancy	14 505	1.03 (0.98-1.07)	.28	0.99 (0.95-1.04)	.68	1.03 (0.98-1.07)	.26
Trimester 1	14 493	1.03 (0.94-1.14)	.50	1.04 (0.94-1.14)	.48	1.11 (1.00-1.22)	.04
Trimester 2	14 503	1.02 (0.97-1.07)	.54	1.00 (0.95-1.05)	.87	1.01 (0.96-1.06)	.69
Trimester 3	13 128	1.12 (1.01-1.25)	.04	0.98 (0.88-1.09)	.66	1.04 (0.93-1.16)	.50
**Pain**							
During pregnancy	14 503	1.05 (1.02-1.09)	.004	1.01 (0.97-1.05)	.58	1.02 (0.99-1.06)	.19
Trimester 1	14 493	1.06 (1.02-1.09)	.001	1.03 (0.99-1.06)	.11	1.01 (0.98-1.05)	.55
Trimester 2	14 503	0.91 (0.82-1.00)	.053	1.01 (0.91-1.11)	.91	0.99 (0.90-1.10)	.90
Trimester 3	13 881	1.03 (1.00-1.07)	.07	1.01 (0.97-1.04)	.73	1.03 (1.00-1.07)	.07
**Migraine**							
Ever	14 493	1.08 (1.03-1.14)	.004	1.05 (1.00-1.11)	.055	0.99 (0.94-1.04)	.65
Before pregnancy	14 539	1.08 (1.02-1.13)	.007	1.05 (1.00-1.11)	.06	0.97 (0.92-1.03)	.34
During pregnancy	14 539	1.12 (1.04-1.20)	.002	1.10 (1.02-1.18)	.009	0.98 (0.92-1.06)	.68
**Depression/anxiety symptoms**							
During pregnancy	14 503	1.15 (1.09-1.22)	5.48 × 10^−7^	1.13 (1.06-1.19)	3.62 × 10^−5^	1.13 (1.07-1.20)	1.71 × 10^−5^
Trimester 1	14 392	1.15 (1.08-1.22)	2.98 × 10^−5^	1.14 (1.07-1.21)	9.10 × 10^−5^	1.12 (1.05-1.20)	4.22 × 10^−4^
Trimester 2-3	13 826	1.15 (1.06-1.24)	8.29 × 10^−4^	1.13 (1.04-1.23)	.002	1.12 (1.03-1.22)	.006
**Depression medication**							
During pregnancy	14 503	1.17 (0.99-1.38)	.06	1.03 (0.87-.21)	.72	1.43 (1.21-1.69)	2.76 × 10^−5^
Trimester 1	14 493	1.16 (0.97-1.39)	.11	1.04 (0.87-1.25)	.65	1.46 (1.21-1.75)	6.47 × 10^−5^
Trimester 2	14 503	1.32 (1.04-1.66)	.02	1.00 (0.79-1.26)	.995	1.32 (1.04-1.68)	.02
**Paracetamol use**							
During pregnancy	14 503	1.03 (1.00-1.07)	.04	1.02 (0.98-1.05)	.31	0.99 (0.95-1.02)	.41
Trimester 1-2	14 493	1.03 (0.99-1.06)	.11	1.01 (0.98-1.05)	.41	0.98 (0.95-1.01)	.24
Trimester 2-3	13 881	1.03 (0.99-1.07)	.19	1.00 (0.96-1.04)	.91	1.00 (0.96-1.04)	.91
**Ibuprofen use**							
During pregnancy	14 503	0.99 (0.93-1.05)	.81	1.06 (1.00-1.12)	.07	0.95 (0.89-1.01)	.08
Trimester 1-2	14 493	0.99 (0.93-1.06)	.85	1.07 (1.00-1.13)	.04	0.96 (0.90-1.02)	.17
Trimester 2-3	13 881	0.94 (0.78-1.13)	.51	0.85 (0.70-1.02)	.09	0.87 (0.72-1.06)	.17

Abbreviations: ADHD, attention-deficit/hyperactivity disorder; OR, odds ratio; PGS, polygenic scores.

^a^ Multiple testing corrected *P* < .002. Trimesters were defined as 0-12 weeks, first trimester; 13-28 weeks, second trimester; and ≥29 weeks, third trimester.

### Sensitivity Analyses

Results using PGSs derived at different *P* value thresholds were consistent with our primary analysis of using a *P* value threshold of less than .05 (eAppendix 9 in the Supplement). Results from inverse probability weighting and multiple imputation analyses were consistent with our primary results using complete case data (eAppendices 3, 5, 6, and 10 in the Supplement).

## Discussion

We examined the association between parental genetic liability to ADHD, autism, and schizophrenia and a wide range of pregnancy-related factors previously observed to be associated with these conditions in offspring. Mothers with a higher ADHD PGS were more likely to be younger at age of childbirth, smoke during pregnancy, have higher BMI, gain more weight during pregnancy, have asthma and depression/anxiety symptoms, and less likely to take folate or other supplements. Our findings were broadly in line with findings from ALSPAC^20^ and the UK Biobank.^28^ Concordant results across these 2 UK-based cohorts represent a cross-cultural replication, strengthening the evidence that some pregnancy-related factors are associated with ADHD genetic liability and emphasizing the need to consider genetic confounding as a potential explanation for parent-offspring associations.

Evidence for an association with PGS does not exclude a causal effect. Genetic liability for neurodevelopmental conditions in parents might increase liability in their offspring through direct genetic effects and increase the likelihood of causal pregnancy-related exposures. Future study designs should attempt to partition genetic confounding from causal effects, triangulating different genetically informative approaches such as within-family Mendelian randomization, sibling comparison, and children-of-twins designs.^32,33,34^ Our findings suggest potential effects of parental genetic liability to ADHD, autism, and schizophrenia on pregnancy-related factors. Even if these pregnancy-related factors are not causal for offspring neurodevelopment, many of them are still known to be causal for other offspring health outcomes (eg, low birth weight^35^). Consequently, future studies should determine whether expecting parents with neurodevelopmental conditions require specific support during pregnancy (eg, to quit smoking and regulate weight gain).

In the case of smoking, studies using different causally informative designs have found that smoking during pregnancy is unlikely to increase the likelihood of ADHD outcomes in offspring via causal mechanisms.^12,36,37,38^ Current findings suggest that ADHD is more likely to increase the risk of smoking during pregnancy, rather than the other way around.

The paternal ADHD PGS association with paternal smoking during pregnancy was of smaller magnitude than the maternal ADHD PGS association with maternal smoking during pregnancy. Thus, for smoking (and caffeine consumption, where disparity was also observed), there are implications for the validity of paternal negative control studies. Such studies assume that associations between maternal and paternal smoking with offspring outcomes will be similarly biased by familial factors such as shared genetics, and therefore any difference between the 2 is likely due to intrauterine effects.^13^ However, we show that associations between maternal and offspring outcomes are more at risk of bias by genetic confounding than associations involving paternal smoking.

Maternal schizophrenia PGS was associated with higher likelihood of smoking and coffee consumption during pregnancy. There is both phenotypic and genetic correlation between coffee consumption and smoking,^39^ making it challenging to account for pleiotropy. Schizophrenia PGS was also associated with lower BMI, consistent with findings from ALSPAC^20^ and the UK Biobank.^28^ We found a novel association between schizophrenia PGS and increased pregnancy weight gain. These findings are important given that exposures such as smoking during pregnancy were thought to play a causal role in schizophrenia.^40^ The findings suggest that observational studies of pregnancy-related exposures and offspring schizophrenia need to be regarded with caution.

Autism PGS, as well as ADHD PGS and schizophrenia PGS, were associated with higher odds of experiencing depression/anxiety symptoms during pregnancy, as reported previously in ALSPAC.^20^ Major depression is also genetically correlated with neurodevelopmental conditions.^41^ The associations we found between schizophrenia PGS and depression/antidepressant medication use in pregnancy highlight the importance of genetically informative designs in studies of prenatal exposure to antidepressants and child neurodevelopment.^42^

There was evidence for an association between ADHD PGS and an increased risk of asthma. Comorbidity between asthma and ADHD has been demonstrated previously.^43,44^ Asthma and ADHD are also genetically correlated,^45^ with ADHD PGS previously found to predict asthma risk in the UK Biobank.^28^ Shared genetic liability between asthma and ADHD could be due to immunological mechanisms as ADHD is also positively associated with other allergic diseases.^46^

There was some weak evidence of ADHD PGS and autism PGS association with migraine. ADHD-migraine comorbidity has been reported in children and adults,^47^ and migraine is genetically correlated with ADHD.^48^ Migraine could represent a mediating or confounding mechanism between the association of ADHD and paracetamol use.^49^

### Strengths and Limitations

Our study has several strengths, including large sample size and availability of many pregnancy-related factors. We were able to compare maternal and paternal PGS associations on the same pregnancy-related factors. We found relatively consistent associations, suggesting that genetic confounding may contribute to some associations between pregnancy-related factors and offspring ADHD, autism, and schizophrenia.

Our study was limited by the small amount of variance explained by neurodevelopmental PGS, especially for autism because of its low common single-nucleotide variant heritability.^16,17,18,50^ Therefore, even where there was no evidence for an association in the current study, it is difficult to exclude associations of small magnitudes. The majority of the prenatal exposure PGS associations identified were of small magnitude. However, given that the PGS only explains a small proportion of the variance in the heritability, these estimates do not capture the full extent of genetic confounding. Consequently, only adjusting for parental PGS in observational studies is unlikely to sufficiently control for genetic confounding. An important next step (when the MoBa offspring are older) is to incorporate offspring phenotypic and genetic information and triangulate different designs to quantify the true extent of genetic confounding in associations between pregnancy exposures and neurodevelopmental outcomes.^51^

We confirmed that ADHD PGS was associated with ADHD behaviors in MoBa mothers and fathers, and previous studies have shown that PGS for ADHD, autism, and schizophrenia predict signs of these conditions in the general population.^29,31,52^ Owing to lack of power, we were not able to investigate other neurodevelopmental conditions, such as Tourette syndrome (cases, n = 4819; controls, n = 9488).^53^ When larger GWAS become available, these investigations can be extended.

We relied on self-reports for many of the pregnancy-related factors. For some exposures (eg, smoking), this might have led to reporting bias. However, results were consistent with paternal exposures during pregnancy, which does not tend to be considered as harmful. Paternal associations of ADHD PGS with smoking were in fact lower than maternal associations, which might indicate that mother’s reporting was not biased by stigma.

As with all cohort studies, MoBa is subject to certain selection biases, for example, underrepresentation of younger parents and those with less education.^23,54,55,56^ Thus, generalizability of results to populations not well-represented in MoBa should not be assumed. However, it is also worth noting that most measures used, and the blood samples from which genotype data arise, were collected during pregnancy, meaning that selective attrition is not a likely source of bias in these results. Genotyping in MoBa prioritized full trios, which likely contributed to differences between the full and genotyped sample. We performed sensitivity analyses using inverse probability weighting and multiple imputation and the results were consistent, suggesting this selection bias did not substantially impact our findings.

## Conclusions

Our study demonstrates associations of ADHD genetic liability with several pregnancy-related factors that have been considered predisposing factors for offspring ADHD. Schizophrenia genetic liability also showed associations with some pregnancy-related factors, including lower prepregnancy BMI, higher pregnancy weight gain, and increased smoking during pregnancy. Autism genetic liability showed few associations with pregnancy-related factors beyond depression. Our findings suggest that pregnant individuals with high ADHD or schizophrenia genetic liability are at increased risk of adverse pregnancy-related exposures. Furthermore, our results indicate that observed associations between asthma, depression, smoking, BMI, pregnancy weight gain, and reduced likelihood of taking supplements with offspring ADHD as well as coffee consumption, smoking, BMI, and higher pregnancy weight gain with schizophrenia in the offspring are likely to be, at least in part, due to shared genetic liability, highlighting the need for genetically informative study designs for causal inference.

## Notes

Supplement. **eAppendix 1.** MoBa genetic data generation and quality control **eAppendix 2.** Polygenic risk score thresholds **eAppendix 3.** Pregnancy-related factors **eAppendix 4.** Principal Component Analysis (PCA) **eAppendix 5.** Inverse Probability Weighting (IPW) **eAppendix 6.** Multiple Imputation (MI) **eAppendix 7.** ADHD symptom measures in adulthood in MoBa **eAppendix 8.** Paternal PGS sample overview and results **eAppendix 9.** PGS associations at different *P* value thresholds **eAppendix 10.** Comparison of complete case, inverse probability weighted and multiple imputation results
eReferences
