# Supplementary material for: Associations Between Pregnancy-Related Predisposing Factors for Offspring Neurodevelopmental Conditions and Parental Genetic Liability to Attention-Deficit/Hyperactivity Disorder, Autism, and Schizophrenia: The Norwegian Mother, Father and Child Cohort Study (MoBa)
Source: JAMA Psychiatry. 2022 Jul 6;79(8):799–810. doi: 10.1001/jamapsychiatry.2022.1728 (PMC9260642; doi:10.1001/jamapsychiatry.2022.1728)
Supplement: Supplement. — eAppendix 1. MoBa genetic data generation and quality control eAppendix 2. Polygenic risk score thresholds eAppendix 3. Pregnancy-related factors eAppendix 4. Principal Component Analysis (PCA) eAppendix 5. Inverse Probability Weighting (IPW) eAppendix 6. Multiple Imputation (MI) eAppendix 7. ADHD symptom measures in adulthood in MoBa eAppendix 8. Paternal PGS sample overview and results eAppendix 9. PGS associations at different P value thresholds eAppendix 10. Comparison of complete case, inverse probability weighted and multiple imputation results eReferences [file jamapsychiatry-e221728-s001.pdf]

Supplemental Online Content

Havdahl A, Wootton RE, Leppert B, et al. Associations between pregnancy-related predisposing factors for offspring neurodevelopmental conditions and parental genetic liability to attention-deficit/hyperactivity disorder, autism, and schizophrenia: the Norwegian Mother, Father and Child Cohort Study (MoBa). *JAMA Psychiatry*. Published online July 6, 2022. doi:10.1001/jamapsychiatry.2022.1728

eAppendix 1. MoBa genetic data generation and quality control

eAppendix 2. Polygenic risk score thresholds

eAppendix 3. Pregnancy-related factors

eAppendix 4. Principal Component Analysis (PCA)

eAppendix 5. Inverse Probability Weighting (IPW)

eAppendix 6. Multiple Imputation (MI)

eAppendix 7. ADHD symptom measures in adulthood in MoBa

eAppendix 8. Paternal PGS sample overview and results

eAppendix 9. PGS associations at different p-value thresholds

eAppendix 10. Comparison of complete case, inverse probability weighted and multiple imputation results

eReferences

This supplemental material has been provided by the authors to give readers additional information about their work.

© 2022 Havdahl A et al. *JAMA Psychiatry.*

eAppendix 1. MoBa genetic data generation and quality control

Genotyping in MoBa is ongoing. For the present study, we used approximately 17,000 trios from the Norwegian Mother, Father and Child cohort, genotyped in three batches. Genotypes were called using GenomeStudio (Illumina, San Diego, USA) and converted to PLINK format files. The first batch, comprising 20,664 individuals and 542,585 SNPs was genotyped at the NTNU Genomics Core Facility (Trondheim, Oslo) using the Illumina HumanCoreExome (Illumina, San Diego, USA) genotyping array, version 12 1.1. The second batch, comprising 12,874 individuals and 547,644 SNPs was genotyped at the NTNU Genomics Core Facility (Trondheim, Oslo) using the Illumina HumanCoreExome (Illumina, San Diego, USA) genotyping array, version24 1.0. The third batch, comprising 17,949 individuals and 692,367 SNPs, was genotyped at ERASMUS MC (the Netherlands) using the Illumina Global Screening Array (Illumina, San Diego, USA) version 24 1.

PLINK version 1.90 beta 3.36 (http://pngu.mgh.harvard.edu/purcell/plink/) was used to conduct the quality control, which has previously been described by Helgeland et al[1]. Known problematic SNPs previously reported by the Cohorts for Heart and Aging Research in Genomic Epidemiology (CHARGE) consortium and Psychiatric Genomics Consortium (PGC) were excluded from each batch. Duplicate samples were removed, and each genotyping batch was split into parents and offspring. Quality control was then conducted by genotyping array in parents and offspring separately.

Individuals were excluded if they had a genotyping call rate below 95% or autosomal heterozygosity greater than four standard deviations from the sample mean. SNPs were excluded if they were ambiguous (A / T and C / G), had a genotyping call rate below 98%, minor allele frequency of less than 1%, or Hardy-Weinberg equilibrium P-value less than 1×10^{-6}. Population stratification was assessed, using the HapMap phase 3 release 3 as a reference, by principal component analysis using EIGENSTRAT version 6.1.4. Visual inspection identified a homogenous population of European ethnicity and individuals of non-European ethnicity were removed. Individuals with a genotyping call rate below 98% or autosomal heterozygosity greater than four standard deviations from the sample mean were then removed. A sex check was done by assessing the sex declared in the pedigree with the genetic sex, which was imputed based on the heterozygosity of chromosome X. When sex discrepancies were identified, the individual was flagged. Relatedness was assessed by flagging one individual from each pairwise comparison of identity-by-descent with a pi-hat greater than 0.1.

The parents and offspring datasets were then merged into one dataset per genotyping batch; keeping only the SNPs that passed quality control in both datasets. All individuals passing the genotyping call rate and autosomal heterozygosity measures were included in the merged datasets. Therefore, the merged datasets included individuals previously excluded or flagged as a duplicate, ethnic outlier, having a sex discrepancy, or high level of relatedness. Concordance checks were then conducted on validated duplicates. Duplicate, tri-allelic and discordant (any discordance between the validated duplicates) SNPs were excluded. Individuals and SNPs with a genotyping call rate below 98% in the merged datasets were excluded. The duplicate sample that was removed before the start of the quality control was then excluded. Mendelian errors identified by the assessment of duos and trios were then recoded to missing. Insertions and deletions were also excluded.

After QC the Human Core Exome 12 batch comprised 20,231 individuals and 384,855 SNPs, the Human Core Exome 24 batch comprised 12,757 individuals and 396,189 SNPs, and the Global Screening Array batch comprised 17,742 individuals and 568,275 SNPs. Phasing was conducted using Shapeit 2 release 837 and the duoHMM approach was used to account for the pedigree structure. Imputation was conducted using the Haplotype reference consortium (HRC) release 1-1 as the genetic reference panel. The Sanger Imputation Server was used to perform the imputation with the Positional Burrows-Wheeler Transform (PBWT). The phasing and imputation were conducted separately for each genotyping batch.

© 2022 Havdahl A et al. *JAMA Psychiatry.*

Post imputation quality control was performed by initially converting the dosages to best-guess genotypes. Individuals were removed if they had a genotyping call rate less than 99% or were of non-European ethnicity. SNPs with an imputation INFO quality score less than 0.8 (in any batch), genotyping call rate less than 98%, minor allele frequency less than 1%, or a Hardy-Weinberg equilibrium P-value less than 1×10^{-6} were removed. Mendelian errors were set to missing. Relatedness, which was accounted for within generation and genotyping batch during pre-imputation QC as described above, was assessed intergenerationally and across batches by flagging one individual from each pairwise comparison of identity-by-descent with a pi-hat greater than 0.15 (excepting known relationships, such as parent-offspring, full-sibling, half-sibling relationships). Individuals were flagged for removal only if the other member of their pair would otherwise be included in the same analysis. One individual from each pair was flagged at random, except when retaining one individual in a pair would keep more duo/trio data intact than the other, in which case the other member was dropped. After quality control, a core homogeneous sample of European ethnicity (based on PCA of markers overlapping with available HapMap markers) individuals across all batches and arrays were available for use in analysis (totals prior to analysis-specific exclusions for relatedness: Nchildren = 15,208; Nmothers = 14,804; Nfathers = 15,198). Additionally, participants were removed from our analysis if they had previously contributed genotype data to either the schizophrenia GWAS (as part of the TOP consortium) or ASD GWAS (as part of the BUPGen consortium).

© 2022 Havdahl A et al. *JAMA Psychiatry.*

eAppendix 2. Polygenic risk score thresholds

eTable 1a. Number of genetic variants (SNPs) included in the polygenic risk scores for attention deficit hyperactivity disorder (ADHD), autism and schizophrenia (SCZ) at specific p-value cut-offs.

**p-value cut-offs**	**N (SNP_{ADHD})**	**N (SNP_{ASD})**	**N (SNP_{SCZ})**
0.0005	1346	1078	2986
0.005	6924	6934	10047
0.05	40056	46381	39731
0.1	67906	81707	61245
0.5	215773	285488	164903

eTable 1b. Correlation matrix of maternal and paternal polygenic risk scores for attention deficit hyperactivity disorder (ADHD), autism and schizophrenia (SCZ), where * = p<0.05.

	**$mPGS_{ADHD}$**	**$mPGS_{ASD}$**	**$mPGS_{SCZ}$**		**$pPGS_{ADHD}$**	**$pPGS_{ASD}$**	**$pPGS_{SCZ}$**
**$mPGS_{ADHD}$**	1			**$pPGS_{ADHD}$**	1		
**$mPGS_{ASD}$**	0.226*	1		**$pPGS_{ASD}$**	0.232*	1	
**$mPGS_{SCZ}$**	0.070*	0.012	1	**$pPGS_{SCZ}$**	0.047*	-0.011	1
**$pPGS_{ADHD}$**	0.003	-0.001	-0.005				
**$pPGS_{ASD}$**	-0.004	0.012	-0.006				
**$pPGS_{SCZ}$**	0.001	-0.003	0.036				

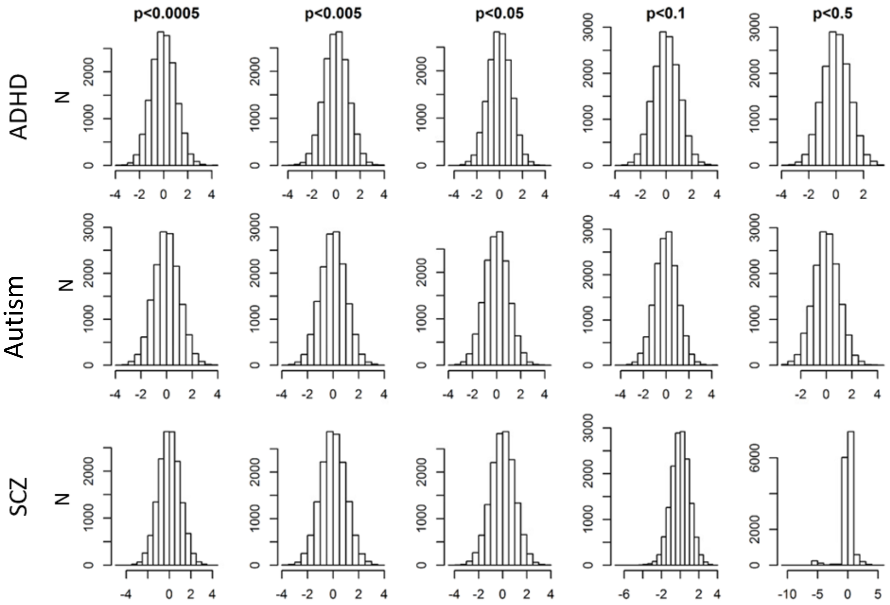

eFigure 1. Histograms of maternal polygenic risk scores (n=13,898) for attention deficit hyperactivity disorder (ADHD), autism and schizophrenia (SCZ) at specific p-value thresholds as indicated on the top.

© 2022 Havdahl A et al. *JAMA Psychiatry.*

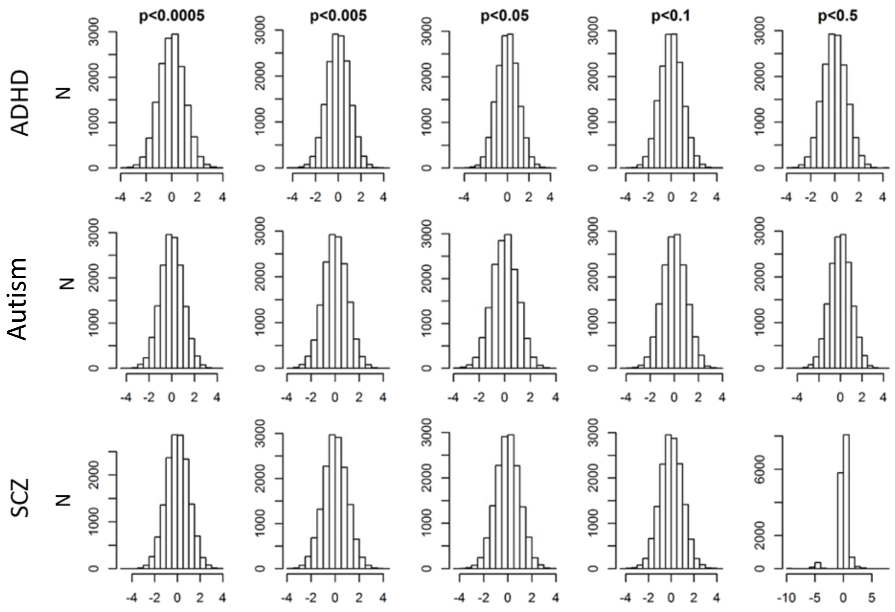

eFigure 2. Histograms of paternal polygenic risk scores (n=13,898) for attention deficit hyperactivity disorder (ADHD), autism and schizophrenia (SCZ) at specific p-value thresholds as indicated on the top.

© 2022 Havdahl A et al. *JAMA Psychiatry.*

eAppendix 3. Pregnancy-related factors

Prenatal factors were chosen after a literature review of potential adverse early life exposures that have previously been reported to be associated with neurodevelopmental conditions. We excluded all pregnancy-related factors with less than 100 cases, given that 100 cases (n_{total} =14,584 mothers) are needed to detect an odds ratio (OR) of 1.3 with 80% power and alpha 0.05. Where information on timing of the measure within pregnancy (trimester) was available, we combined them into one “during pregnancy” variable.

Phenotypes related to maternal behaviour and lifestyle during pregnancy included maternal age at delivery[2], cigarette smoking[3,4], alcohol consumption[5], binge drinking[6], coffee drinking and binge coffee drinking[7], intake of nutritional supplements and intake of folate supplementation before[8–10] and during pregnancy.

Phenotypes related to physical health during pregnancy included pre-pregnancy BMI and weight gain during pregnancy[11,12], type II diabetes (T2D) (incl. gestational diabetes)[13], high blood pressure (incl. preeclampsia)[13,14], vaginal bleeding[15–17], anaemia[9,18], fever[19], upper and lower respiratory infections, urinary tract infections (UTI), any infection or inflammation[20–22], B12 insufficiency[23], type I diabetes (T1D), hypo-/hyperthyroidism[5], asthma[20], psoriasis and other autoimmune diseases[20,24–27].

Self-reported indicators of maternal medication use during pregnancy included medication use for depression[5,28], depression or anxiety, epilepsy, pain (paracetamol and ibuprofen specifically), headache or migraine and fever[5,29–33].

A detailed list of sources for each pregnancy-related factor can be found below (Tables S2-S4). Unless otherwise specified, measures occurred during pregnancy. If the question was asked at multiple time points, then individuals were coded as 1 if they endorsed the measure at anytime point during pregnancy. For trimester specific factors we used the self-report from the respective questionnaires. Two time periods had information available in two consecutive questionnaires, which are i) “13+week” in questionnaire 1 and “13-16 week” in questionnaire 3 and ii) “29+ weeks” in questionnaire 3 and “last part of pregnancy” in questionnaire 4. To address this overlap, we collapsed both questions into one category and assigned them to the second and third trimester, respectively. Trimesters were defined as 0-12 weeks = first trimester, 13-28 weeks = second trimester and 29 + weeks = third trimester.

Information about pregnancy-related factors in mothers and fathers was obtained from self-reported questionnaire data and the Norwegian birth registry. For Tables S2-S4, self-report questionnaires are indicated as Q1, Q3, Q4 and QF. On average, mothers responded to Q1 at 15 weeks of gestation, Q3 at 30 weeks gestation and Q4 at 6 months after birth. Fathers responded to QF which was sent out alongside the 15-week questionnaire to mothers. Items from the Medical Birth Registry of Norway are indicated as MBRN.

eTable 2. Maternal variable definitions for primary analysis

	Source	Description
**Behaviour and lifestyle**		
**Maternal age**	**MBRN**<br>MORS_ALDER	Maternal age in years from the medical birth registry. Mothers were set to missing if age was greater than 60 years.
**Cigarette smoking**	**Q1**<br>AA1356<br>**Q3**<br>CC1037<br>**Q4**<br>DD732	Cigarette smoking at any time during pregnancy coded as 1 if any of the items below were 1, else 0. Mothers were asked:<br>Q1: "Do you smoke now (after you became pregnant)?"<br>Q3: "Do you smoke at present?"<br>Q4: "What were your smoking habits during the last 3 months of your pregnancy?"<br>For all 3 items, response categories were "1-No", "2-Sometimes" and "3-Daily". If they responded no, this was coded as 0 (non-smoker) and both sometimes and daily were coded as 1 (smoker).
**Alcohol consumption**	**Q1**<br>AA1454<br>**Q3**<br>CC1157<br>CC1158 CC1159<br>**Q4**<br>DD774	Alcohol consumption at any time during pregnancy, coded as 1 if any of the items below were 1, else 0. Mothers were asked how often they had consumed alcohol in:<br>Q1: "During pregnancy"<br>Q3: "In this pregnancy, week 0-12";" In this pregnancy, week 13-24"; "In this pregnancy, week 25+"<br>Q4: "Last 3 months of pregnancy"<br>Response categories for all items were on a 7-point scale, from "1-Approximately 6-7 time a week" to "7-Never". Responses of never were coded as 0 (never) and all other responses were coded as 1 (ever).
**Binge drinking**	**Q1**<br>AA1463<br>**Q3**<br>CC1161 CC1162<br>CC1163	Binge drinking at any time during pregnancy, coded as 1 if any of the items below were coded 1, else 0.<br>Mothers were asked "Did you drink 5 units or more at least once…"<br>Q1: "…during pregnancy".<br>Q3: "…in this pregnancy, week 0-12"; "…in this pregnancy, week 13-24"; "…in this pregnancy, week 25+".<br>Response categories of "Several times per week/Once a week/1-3 times a month/Less than once a month" were recoded as 1=ever and "Never" was recoded as 0.
**Coffee consumption**	**Q1**<br>AA1378 AA1381<br>AA1384<br>**Q3**<br>CC1119 CC1121<br>CC1123	Any coffee consumption at any time during pregnancy, coded 1 if any of the below were coded 1, else 0. Mothers were asked: "What was your fluid consumption (number of cups/glasses) per day during pregnancy?"<br>Categories of coffee were "Filter coffee", "Instant coffee" and "Boiled coffee" were 1 cup of more =1 (ever) and 0 cups =0 (never).
**Binge coffee drinking**	**Q1**<br>AA1378 AA1381<br>AA1384<br>**Q3**<br>CC1119 CC1121<br>CC1123	Binge drinking of coffee at any point during pregnancy. Coded 1 if any of the coffee consumption variables (described above) were reported as 6 or more cups of coffee per day at any of the time points.
**No supplements taken**	**Q1** AA939<br>**Q3** CC770	Supplements taken during pregnancy, coded as 1 if no supplements were taken either at Q1 or Q3 below, else 0.<br>Mothers were asked:<br>Q1: "Do you take vitamins, minerals or other dietary supplements?" (yes=0, no=1)<br>Q3: "Have you taken vitamins, minerals or other nutritional supplements after the 13th week of pregnancy?" (yes=0, no=1)
**Folate supplement before pregnancy**	**Q1**<br>AA940 AA941<br>AA942	Folic acid supplements taken at any point in the 6 months prior to pregnancy, coded as 1 if participant endorsed taking folic acid in any of weeks 26-9, 8-5 or 4-0 before the pregnancy.
**Folate supplement during pregnancy**	**Q1**<br>AA943 AA944<br>AA945 AA946	Folic acid supplements taken at any point during the pregnancy, coded as 1 if participant endorsed taking folic acid in pregnancy, in either weeks 0-4, 5-8, 9-12, or 13+.
**Metabolic Conditions**		
**BMI before pregnancy**	**Q1** AA87 AA85	Body mass index (BMI) calculated as (kg weight/(m height*m height)).<br>How tall are you? Reported in cm.<br>What did you weigh at the time you became pregnant? Reported in kg.

© 2022 Havdahl A et al. *JAMA Psychiatry.*

		Individuals shorter than 100cm were excluded. Individuals weighing less than 35 kg or greater than 200kg were excluded.
**Weight gain**	**Q1** AA85<br>**Q4** DD672	Total weight gained during the pregnancy measured in kilograms (kg). Calculated as weight at end of pregnancy (Q4) minus weight when mother became pregnant (Q1). Individuals weighing less than 35 kg or greater than 200kg either before or after were excluded.
**Type 2 diabetes (incl. gestational diabetes)**	**Q1** AA510<br>AA519<br><br>**MBRN**<br>DIABETES_<br>MELLITUS	Overall diabetes during pregnancy, combining both gestational diabetes and type 2 diabetes, coded as 1 indicates diabetes and 0 none.<br>Questionnaire variables were:<br>Diabetes during pregnancy, treated with insulin (ticked yes vs no)<br>Diabetes during pregnancy, not treated with insulin (ticked yes vs no)<br>Answering yes to either of the above or have a value of 2/3/4 for the MBRN DIABETES_MELLITUS variable, where:<br>2 = diabetes type2<br>3 = diabetes (not type 1 or type 2 but unspecified)<br>4 = gestational diabetes
**High blood pressure in pregnancy (incl. preeclampsia)**	**Q1** AA555<br>**Q3** CC113<br>**Q4** DD434<br>**MBRN**<br>PREEKL	Coded 1 if any of the below self-reported high blood pressure were coded 1, or if the MBRN had registered preeclampsia.
**Hyper-/Hypothyroidism**	**Q1** AA564	Hypothyroidism or hyperthyroidism during pregnancy (yes/no), where yes is coded as 1 and no as 0.
**Infectious and autoimmune diseases**		
**Upper respiratory tract infections**	**Q1** AA356-AA359<br>AA366-AA369<br>**Q3** CC508-CC512<br>CC520-CC524<br>**Q4** DD490	Tonsilitis, sinusitis and/or otitis during pregnancy (tick “yes”) on any of the variables below.
**Lower respiratory tract infections**	**Q1** AA386-AA389<br>**Q3** CC544-CC548<br>**Q4** DD498	Pneumonia and/or bronchitis during pregnancy (tick “yes”) on any of the variables below.
**Urinary tract infection**	**Q1** AA789<br>**Q3** CC592-CC596	Do you have or have you had any of the following illnesses or health problems? (ticked “yes” to UTI at any of the during pregnancy time points)
**Fever**	**Q1** AA326-AA329<br>AA336-AA339<br>**Q3** CC712-CC716<br>**Q4** DD554	Fever endorsed at any time during pregnancy, including fever with a rash and a fever of over 38 C. Coded as 1 if endorsed at any time during pregnancy and 0 if not endorsed during pregnancy.
**Asthma**	**Q1** AA420<br>**Q3** CC640-CC644	Asthma reported at any time during pregnancy (tick “yes” on any of the time points during pregnancy), where any yes is coded as 1 and no is coded as 0.
**Psoriasis**	**Q1** AA474	Psoriasis in pregnancy (tick “yes”) coded as 1 for yes and 0 for no.
**Type 1 diabetes**	**MBRN**<br>DIABETES_MELLITUS	Type 1 diabetes during pregnancy, as indicated by the medical birth registry. If the participant was coded as 1 in MBRN, this indicated type 1 diabetes, all other responses were recoded to 0 (no type 1 diabetes).
**Other autoimmune disease**	**Q1**<br>AA645 AA654<br>AA843 AA618<br>AA627	Other autoimmune disease in pregnancy (tick “yes” for any of the variables below – arthritis, lupus (SLE), multiple sclerosis, celiac disease, Chron’s disease). Coded as 1 if they responded yes to any of these variables.
**Other physical health conditions**		
**Vaginal bleeding**	**MBRN**<br>BLODN_F13<br>BLODN_13_28<br>BLODN_E28	Bleeding in pregnancy either before week 13 or between week 13 and 28, as recorded in the medical birth registry
**B12 insufficiency**	**Q1** AA582	B12 insufficiency in pregnancy (tick “yes”)

© 2022 Havdahl A et al. *JAMA Psychiatry.*

**Anaemia/low haemoglobin in early pregnancy**	**Q1** AA573	Anaemia/low haemoglobin in pregnancy (tick "yes")
**Indication for medicine use**		
**Lifetime depression**	**Q1** AA1572-AA1577	Coded as 1 if mothers fulfilled criteria on the Lifetime History of Major Depression scale (Kendler et al., 1993). Overall question: "Have you ever experienced the following for a period of 2 weeks or more?" Ticked "yes" for symptom *Felt depressed* + at least two other depression symptoms simultaneously (*problems with appetite/eating too much, bothered by lack of energy, blamed self and felt worthless, problems with concentrations or making decisions*).
**Depression/Anxiety**	**Q1** AA1548-AA1552 **Q3** CC1202-CC1209	Symptoms of depression/anxiety during pregnancy. Coded as 1 if mothers reported depression and anxiety symptoms on the Symptoms Checklist (SCL) short form at or above the recommended mean item score cut-off of 2, at either Q1 or Q3 [within the last 2 weeks]. SCL-5 reference Strand, B.H., Dalsgard, O.S., Tambs, K., & Rognerud, M. 2003. SCL-8 reference: Tambs, K. & Røysamb E. 2014.
**Med Depression**	**Q1** AA873-AA876 **Q3** CC682-CC686	Having taken medication for depression during pregnancy, coded as 1 if responded yes at any time point during pregnancy, and 0 if they were not taking medication during pregnancy. Mothers were instructed that medication included: "… taken medication (tablets, mixtures, suppositories, inhalers, creams, etc.) in conjunction with the illness or health problem…".
**Med Depression or anxiety**	**Q1** AA873-AA876 AA882-AA885 **Q3** CC682-CC686	Having taken medication for depression or anxiety, combination of the medication for depression variable described above and if they endorced having taken medication for anxiety at any of the pregnancy timepoints, coded as 1 for either depression or anxiety and 0 for neither.
**Pain**	**Q1** AA176-AA179 AA186-AA189 AA196-AA199 AA206-AA209 **Q3** CC340-CC344 CC352-CC356 CC364-CC368	Any pain experienced during pregnancy, other than headache and migraine. Types of pain included: pelvic relaxation/girdle pain, abdominal pain, back pain, neck and shoulder pain, and other pains in muscles/ joints. Participants are coded as 1 if they endorsed any of these types of pain at any stage of pregnancy.
**Migraine**	**Q1** AA816	Endorsing having had a migraine during pregnancy so far.
**Headache**	**Q1** AA825	Endorsed having had a headache during pregnancy so far.
**Epilepsy**	**Q1** AA834	Endorsed having had epilepsy during pregnancy so far.
**Med Pain**	**Q1** AA185 AA195 AA205 AA215 **Q3** CC351 CC363 CC375	Having reported taking medication for any of the following types of pain during pregnancy: pelvic relaxation/girdle pain, abdominal pain, back pain, neck and shoulder pain, and other pains in muscles/ joints. Individuals who took medication for >=1 day were coded as 1 and 0 days as 0.
**Paracetamol**	Medication names reported in **Q1/Q3/Q4** converted to ATC codes	Any ATC (the Anatomical Therapeutic Chemical classification system) codes that indicate the mother was taking paracetamol during pregnancy (combined Q1/Q3/Q4). ATC codes are generated from free text responses to "name of medication". Paracetamol codes included: N02BE01-, N02BE51-, N02BE71-, N02BE01- M01AE01-, N02BE51- M01AE01-, N02BE01- M01AE51-, N02BE51- M01AE51-
**Ibuprofen**	Medication names reported in **Q1/Q3/Q4** converted to ATC codes	Any ATC (the Anatomical Therapeutic Chemical classification system) codes that indicate the mother was taking ibuprofen during pregnancy (combined Q1/Q3/Q4). ATC codes are generated from free text responses to "name of medication". Ibuprofen codes included: M01AE01-, M01AE51-
**Med Fever**	**Q1** AA335 AA345	Having endorsed taking medication for a fever with rash or a fever over 38 C.
**Med Pain or fever**	**Q1** AA185 AA195 AA205 AA215 A335 AA345 **Q3** CC351 CC363 CC375	Having endorsed taking medication for a fever with rash, fever over 38 C, pelvic relaxation/girdle pain, abdominal pain, back pain, neck and shoulder pain, and other pains in muscles/ joints.

© 2022 Havdahl A et al. *JAMA Psychiatry.*

eTable 3. Maternal variable definitions for secondary analysis: Timing during gestation

	Source	Description
**Cigarette Smoking**		
**Ever**	**Q1** AA1355	Have you ever smoked? (yes (1) vs no (0))
**During pregnancy**	**Q1** AA1356<br>**Q3** CC1037<br>**Q4** DD732	Cigarette smoking either at T1, T2 or T3 (1) vs neither (0), as described below.
**Trimester 1**	**Q1** AA1356	Do you smoke now (after you became pregnant)? (Sometimes/Daily vs No)
**Trimester 2**	**Q3** CC1037	Do you smoke at present? (Sometimes/Daily vs No)
**Trimester 3**	**Q4** DD732	What were your smoking habits during the last 3 months of your pregnancy? (Smoked sometimes/Smoked daily vs Didn't smoke)
**Alcohol consumption**		
**Ever**	**Q1** AA1452	Have you ever consumed alcohol? (yes vs no)
**During pregnancy**	**Q1** AA1454<br>**Q3** CC1157<br>CC11159<br>CC1158<br>**Q4** DD774	Alcohol consumption either at T1, T2 or T3 (1) vs neither (0), as described below.
**Trimester 1**	**Q1** AA1454<br><br>**Q3** CC1157	How often do you consume alcohol during the pregnancy [so far]? (ever [response categories for 6 frequencies from less than once a month to 6-7 times a week] vs never)<br>How often did you consume alcohol in this pregnancy, week 0-13? (ever [response categories for 6 frequencies from less than once a month to 6-7 times a week] vs never)
**Trimester 2**	**Q3** CC1158	How often did you consume alcohol in this pregnancy, week 13-24? (ever [response categories for 6 frequencies from less than once a month to 6-7 times a week] vs never)
**Trimester 3**	**Q4** DD774	How often did you drink alcohol during the last 3 months of your pregnancy? (ever [response categories for 6 frequencies from less than once a month to 6-7 times a week] vs never)
**High blood pressure**		
**Before pregnancy**	**Q3** CC117<br>(hiBPQ3_1)	High blood pressure before pregnancy (yes vs no) "Have you had high blood pressure without being pregnant?"
**During pregnancy (incl. preeclampsia)**	**Q1** AA555<br>**Q3** CC113<br>**Q4** DD434<br>**MBRN**<br>PREEKL	Coded 1 if any of the below self-reported high blood pressure were coded 1, or if the MBRN had registered preeclampsia.
**Trimester 1**	**Q1** AA555	High blood pressure during pregnancy (ticked "yes")
**Trimester 2**	**Q3** CC113	Has the midwife or doctor told you that you have or have had high blood pressure during this pregnancy? (yes vs no)
**Trimester 3**	Q4 DD434	High blood pressure during last trimester (ticked "yes")
**Vaginal bleeding**		
**During pregnancy**	**MBRN**<br>BLODN_F13<br>BLODN_13_28<br>BLODN_E28	Bleeding in pregnancy either before week 13 or between week 13 and 28, as recorded in the medical birth registry
**Trimester 1**	**MBRN**<br>BLODN_F13	Bleeding in pregnancy before week 13
**Trimester 2**	**MBRN**<br>BLODN_13_28	Bleeding in pregnancy between week 13 and 28
**Trimester 3**	**MBRN**<br>BLODN_E28	Bleeding in pregnancy after week 28
**Urinary tract infections**		

© 2022 Havdahl A et al. *JAMA Psychiatry.*

**Before pregnancy**	**Q1** AA788	Do you have or have you had any of the following illnesses or health problems? [Before Pregnancy] (ticked "yes" to UTI)
**During pregnancy**	**Q1** AA789<br>**Q3** CC592-CC596	Do you have or have you had any of the following illnesses or health problems? (ticked "yes" to UTI at any of the during pregnancy time points)
**Trimester 1**	**Q1** AA789	Urinary tract infections during pregnancy [so far] (ticked "yes")
**Trimester 2**	**Q3** CC592-CC595	Bladder infection/cystitis (ticked "yes" for either pregnancy week 13-16, 17-20, 21-24, 25-28)
**Trimester 3**	**Q3** CC596	Bladder infection/cystitis (ticked "yes" for either pregnancy week 29+)
**Fever**		
**During pregnancy**	**Q1** AA326-AA329<br>AA336-AA339<br>**Q3** CC712-CC716<br>**Q4** DD554	Fever endorsed at any time during pregnancy
**Trimester 1**	**Q1** AA326-AA328<br>AA336-AA338	Fever with rash (ticked "yes" for either pregnancy weeks 0-4, 5-8, 9-12)<br>Fever over 38 C (ticked "yes" for either pregnancy weeks 0-4, 5-8, 9-12)
**Trimester 2**	**Q1** AA329 AA339<br>**Q3** CC712-CC715	Fever with rash or Fever over 38 C (ticked "yes" for pregnancy week 13+)<br>Fever (ticked "yes" for either pregnancy weeks 13-16, 17-20, 21-24, or 25-28)
**Trimester 3**	**Q3** CC716<br>**Q4** DD554	Fever (ticked "yes" for pregnancy week 29+)<br>Fever (ticked "yes" for last trimester)
**Pain**		
**During pregnancy**	**Q1** AA176-AA179<br>AA186-AA189<br>AA196-AA199<br>AA206-AA209<br>**Q3** CC340-CC344<br>CC352-CC356<br>CC364-CC368	Any pain experienced during pregnancy, other than headache and migraine. Types of pain included: pelvic relaxation/girdle pain, abdominal pain, back pain, neck and shoulder pain, and other pains in muscles/ joints. Participants are coded as 1 if they endorsed any of these types of pain at any stage of pregnancy.
**Trimester 1**	**Q1** AA176-AA178<br>AA186-AA188<br>AA196-AA198<br>AA206-AA208	Endorsing pelvic relaxation, abdominal pain, back pain or neck and shoulder pain during weeks 0-4/5-8/9-12 of pregnancy.
**Trimester 2**	**Q1** AA179 AA189<br>AA199 AA209<br>**Q3** CC340-CC343<br>CC352-CC355<br>CC63-CC367	Endorsing pelvic relaxation, abdominal pain, back pain or neck and shoulder pain during weeks 13+ of pregnancy.<br>Endorsing pelvic girdle pain, back pain, or other pains in muscles/ joints during weeks 13-16/17-20/21-24/25-28 of pregnancy.
**Trimester 3**	**Q3** CC344 CC356<br>CC368	Endorsing pelvic girdle pain, back pain, or other pains in muscles/ joints during weeks 29+ of pregnancy.
**Migraine**		
**Ever**	**Q1** AA815 AA816	Having endorsed either having a migraine before pregnancy or during pregnancy.
**Before pregnancy**	**Q1** AA815	Endorsing having had a migraine before pregnancy.
**During pregnancy**	**Q1** AA816	Endorsing having had a migraine during pregnancy so far.
**Depression/anxiety symptoms**		
**During pregnancy**	**Q1** AA1548-AA1552<br>**Q3** CC1202-CC1209	Symptoms of depression/anxiety during pregnancy. Coded as 1 if mothers reported depression and anxiety symptoms on the Symptoms Checklist (SCL) short form at or above the recommended mean item score cut-off of 2, at either Q1 or Q3 [within the last 2 weeks]. SCL-5 reference Strand, B.H., Dalsgard, O.S., Tambs, K., & Rognerud, M. 2003. SCL-8 reference Tambs, K. & Røysamb E. 2014.
**Trimester 1**	**Q1** AA1548-AA1552	
**Trimester 2-3**	**Q3** CC1202-CC1209	
**Medication for depression**		
**During pregnancy**	**Q1** AA873-AA876<br>**Q3** CC682-CC686	
**Trimester 1**	**Q1** AA873 AA874<br>AA875	If you have used tablets, mixtures, suppositories, inhalers, creams, etc. in connection with the illness [depression], if endorsed for any of the time points during T1 (0-4/5-8/9-12 weeks).

© 2022 Havdahl A et al. *JAMA Psychiatry.*

**Trimester 2**	**Q1** AA876<br>**Q3** CC682 CC683 CC684 CC685	If you have used tablets, mixtures, suppositories, inhalers, creams, etc. in connection with the illness [depression], if endorsed for any of the time points during T2 (13+/13-16/17-20/21-24/25-28)
**Trimester 3**	**Q3** CC686	If you have used tablets, mixtures, suppositories, inhalers, creams, etc. in connection with the illness [depression], if endorsed at 29+ weeks.
**Paracetamol**		
**During pregnancy**	Medication names reported in Q1/Q3 converted to ATC codes	Any ATC (the Anatomical Therapeutic Chemical classification system) codes that indicate the mother was taking paracetamol during pregnancy (combined Q1/Q3). Paracetamol codes included: N02BE01-, N02BE51-, N02BE71-, N02BE01- M01AE01-, N02BE51- M01AE01-, N02BE01- M01AE51-, N02BE51- M01AE51-
**Trimester 1-2**	**Q1** Medication codes	ATC codes indicating paracetamol use at Q1.
**Trimester 2-3**	**Q3** Medication codes	ATC codes indicating paracetamol use at Q3.
**Ibuprofen**		
**During pregnancy**	Medication names reported in Q1/Q3 converted to ATC codes	Any ATC (the Anatomical Therapeutic Chemical classification system) codes that indicate the mother was taking ibuprofen during pregnancy (combined Q1/Q3). Ibuprofen codes included: M01AE01-, M01AE51-
**Trimester 1-2**	**Q1** Medication codes	ATC codes indicating ibuprofen use at Q1.
**Trimester 2-3**	**Q3** Medication codes	ATC codes indicating ibuprofen use at Q3.

© 2022 Havdahl A et al. *JAMA Psychiatry.*

eTable 4. Paternal variable definitions

	Source	Description
**Paternal age**	**MBRN** FARS_ALDER	Paternal age in years from the medical birth registry. Age was set to missing if fathers were older than 70 years.
**Ever smoked**	**QF** FF214	Have you ever smoked? Coded as Yes = 1 and No = 0.
**Cigarette smoking**	**QF** FF218	Smoking during the partner's pregnancy. Fathers were asked: "Do you smoke now after your partner became pregnant?". Coded as No=0 and Yes, sometimes/Yes, daily = 1.
**Ever drank alcohol**	**QF** FF242	Have you ever drunk alcohol? Coded as Yes = 1 and No = 0.
**Binge drinking before pregnancy**	**QF** FF473	Reporting binge drinking before the partner's pregnancy. Fathers were asked: "Have you drunk 5 alcohol units or more on at least one occasion in the six months before your partner became pregnant?". There were 5 response categories: Never = 0, Several times per week/Once per week/1-3 times per month/Less than once per month = 1.
**Binge drinking**	**QF** FF474	Reporting binge drinking during the partner's pregnancy. Fathers were asked: "Have you drunk 5 alcohol units or more on at least one occasion now after your partner became pregnant?". There were 5 response categories: Never = 0, Several times per week/Once per week/1-3 times per month/Less than once per month = 1.
**Coffee consumption**	**QF** FF424 - FF426	Fathers were asked "How often do you drink the following?". Coffee categories were: "Filter-and instant coffee"/"Boiled/Cafeteria coffee"/ "Other coffee, espresso or similar". There were 5 response categories: Seldom/never =0 and 1-6 glasses per week/1 glass per day/2-3 glass per day/4 glasses or more per day = 1. If fathers had a 1 for any of the three coffee types, this was an overall code of 1.
**Binge coffee drinking**	**QF** FF424 - FF426	If fathers reported drinking 4 glasses or more per day of any of the three coffee types ("Filter-and instant coffee"/"Boiled/Cafeteria coffee"/ "Other coffee, espresso or similar") then they were coded as 1 for binge coffee drinking. Lower levels of consumption were all coded as 0.
**No supplements taken**	**QF** FF452	"Do you use any form of dietary supplement?" (No = 1/Yes = 0)
**Paternal illness and conditions**		
**BMI**	**QF** FF333 FF334	"How tall are you?" [in cm]<br>"How much do you weigh?" [in kg]<br>Individuals shorter than 100cm were excluded. Individuals weighing less than 35 kg or greater than 200kg were excluded.<br>Body mass index (BMI) calculated as (weight [kg]/height [m]^2).
**Type 2 diabetes (incl. gestational diabetes)**	**QF** FF149	"Do you have, or have you had any of the following illnesses or health problems?" Ticked "yes" to "Diabetes".
**High blood pressure**	**QF** FF173	"Do you have, or have you had any of the following illnesses or health problems?" Ticked "yes" to "High blood pressure".
**Asthma**	**QF** FF122	"Do you have, or have you had any of the following illnesses or health problems?" Ticked "yes" to "Asthma".
**Psoriasis**	**QF** FF128	"Do you have, or have you had any of the following illnesses or health problems?" Ticked "yes" to "Psoriasis".
**Other autoimmune disease**	**QF** FF143 FF170	"Do you have, or have you had any of the following illnesses or health problems?" Ticked "yes" to either "Crohn's disease/ulcerative colitis (diarrhoea, constipation intermittent pain)" or "Bechterew's disease/ rheumatoid arthritis"
**Pain**	**QF** FF161 FF164 FF167	"Do you have, or have you had any of the following illnesses or health problems?" Ticked "yes" to either "Repeated neck and shoulder pain", "Lower back pain" or "Prolonged muscle pain".
**Headache**	**QF** FF137	"Do you have, or have you had any of the following illnesses or health problems?" Ticked "yes" to "Other frequent headaches". [As opposed to migraines].
**Migraine**	**QF** FF134	"Do you have, or have you had any of the following illnesses or health problems?" Ticked "yes" to "Migraine".

© 2022 Havdahl A et al. *JAMA Psychiatry.*

**Lifetime depression**	**QF** FF259-FF264	Lifetime History of Major Depression as assessed with items relating closely to DSM-III criteria (Kendler et al., 1993). Overall question: "Have you ever experienced the following for a period of 2 weeks or more?" Ticked "yes" for symptom *Felt depressed* + at least two other depression symptoms simultaneously (*problems with appetite/eating too much, bothered by lack of energy, blamed self and felt worthless, problems with concentrations or making decisions*).

© 2022 Havdahl A et al. *JAMA Psychiatry.*

eAppendix 4. Principal Component Analysis (PCA)

A Bonferrroni correction for multiple testing would be too conservative for our analyses given the high correlation between variables, and would result in an overall alpha <5% and an inflated rate of false negative findings. Therefore, to correct for multiple testing of the 37 pregnancy-related factors, while accounting for the correlations between them, the number of independent tests was determined based on the number of principal components that explained more than 80% of covariance between the pregnancy-related factors in a principal component analysis (PCA). The 80% PCA approach was chosen to keep the overall alpha as close to 5% as possible.

An unrotated PCA was performed using the pca function in STATA 16.1 (see Table S2) and the number of PCs that explained 80% of covariance between the pregnancy-related factors was declared as independent tests. We concluded that 25 independent tests were performed in our main analysis, resulting in a multiple testing corrected p-value of p<0.002 (0.05/25). A conservative Bonferroni correction would yield a multiple testing corrected p-value of p<0.001 (0.05/37), assuming all tests to be independent.

eTable 5. Unrotated principal component analysis of 37 pregnancy related factors in 14,539 mothers of MoBa

	Eigenvalue	Cumulative %		Eigenvalue	Cumulative %
Comp1	2.32	0.06	Comp20	0.96	0.69
Comp2	1.93	0.11	Comp21	0.94	0.72
Comp3	1.83	0.16	Comp22	0.92	0.74
Comp4	1.60	0.21	Comp23	0.91	0.77
Comp5	1.41	0.25	Comp24	0.87	0.79
Comp6	1.38	0.28	Comp25	0.86	0.81
Comp7	1.35	0.32	Comp26	0.84	0.83
Comp8	1.23	0.35	Comp27	0.82	0.86
Comp9	1.19	0.39	Comp28	0.77	0.88
Comp10	1.16	0.42	Comp29	0.75	0.90
Comp11	1.13	0.45	Comp30	0.70	0.92
Comp12	1.05	0.48	Comp31	0.69	0.94
Comp13	1.02	0.50	Comp32	0.64	0.95
Comp14	1.02	0.53	Comp33	0.61	0.97
Comp15	1.01	0.56	Comp34	0.51	0.98
Comp16	1.00	0.58	Comp35	0.50	1.00
Comp17	1.00	0.61	Comp36	0.10	1.00
Comp18	0.98	0.64	Comp37	0.01	1.00
Comp19	0.96	0.66			

eAppendix 5. Inverse Probability Weighting (IPW)

Genetic liability for ADHD and schizophrenia have been shown to be associated with attrition in other studies and genotyping in MoBa was originally performed on a selected subset of participants, for whom DNA samples of full mother, father, child trios was available. A comparison of the not genotyped and genotyped sample of MoBa suggested that the samples differed according to many analysed factors. Therefore, we performed IPW on missing maternal genetic data to adjust for this sampling bias (n = 80,922 missing genotype data, n = 12,564 complete genotype data). The prediction model for missingness was built using a logistic lasso approach (lassologit function from package lassopack[1,2] in STATA 13), selecting the best fitting model (based on Bayesian Information Criterion (BIC)) from a set of 233 variables from birth registry data and MoBa questionnaire 1 at study recruitment. All prediction variables had less than 10% missing data. Weights were derived from the selected model including 32 variables (Table S3), which predicted missingness with a pseudo R^2 = 0.016. Weights ranged from 3.39 to 136.05. A sensitivity analysis using stabilised weights (and all weights greater than 10 set to 10) revealed no change to the pattern of results. Results of the IPW analysis are compared with the complete case results in Supplementary Figure S4.

eTable 6. Model used to derive inverse probability weights for missingness of genotype of MoBa mothers to account for sampling bias (n=93,486, pseudo R^2=0.016)

	**Log(OR)**	**SE**	**p-value**
APGAR 5min	-0.035	0.016	0.034
APGAR 1min	-0.037	0.012	0.002
Gestational length	-0.051	0.007	<0.001
Birthweight	-0.0001	2E-05	<0.001
Paternal age at birth	0.016	0.002	<0.001
Never been forced to have sexual intercourse	-0.655	0.043	<0.001
Never having taken ecstasy	-0.061	0.031	0.051
Living with parents	0.272	0.099	0.006
Living with a spouse/partner	-0.473	0.060	<0.001
Other long illnesses or health problems before pregnancy	-0.104	0.041	0.012
Depression during pregnancy	0.174	0.073	0.018
Urinary tract infections before pregnancy	-0.051	0.023	0.028
Kidney infection/ pyelonephritis before pregnancy	-0.097	0.050	0.052
Other gastrointestinal problems before pregnancy	-0.141	0.045	0.002
Duodenal/stomach ulcer before pregnancy	0.310	0.134	0.020
Hypothyroidism or hyperthyroidism during pregnancy	0.168	0.076	0.028
Herpes (cold sores) before pregnancy	-0.077	0.029	0.008
Sugar in urine (weeks 9-12)	0.331	0.140	0.018
Fever with rash (weeks 0-4)	1.584	0.720	0.028
Oedema during pregnancy (weeks 13+)	0.158	0.054	0.004
Unusual tiredness/sleepiness (weeks 9-12)	-0.034	0.024	0.158
Unusual tiredness/sleepiness (weeks 5-8)	-0.070	0.024	0.004
Constipation during pregnancy (weeks 5-8)	-0.043	0.025	0.084
Itchy in pregnancy (weeks 0-4)	0.265	0.115	0.021
Nausea with vomiting (weeks 0-4)	0.091	0.039	0.018
Abdominal pain (weeks 5-8)	-0.079	0.029	0.008
Pelvic pain (weeks 13+)	0.080	0.034	0.019
Offspring congenital malformations	0.149	0.049	0.002
Caesarean delivery	0.409	0.055	<0.001
Spontaneous delivery	-0.105	0.029	<0.001

Vaginal bleeding during pregnancy (before week 13)	-0.189	0.055	0.001
Vaginal bleeding during pregnancy (week 13-28)	0.196	0.085	0.021

eTable 7. Inverse Probability Weighted Associations between Polygenic Scores and Exposures during Pregnancy

		PGS ADHD			PGS Autism			PGS Schizophrenia		
	N	OR	95% CI	p	OR	95% CI	p	OR	95% CI	p
**Behavior and lifestyle**										
Maternal age*	14394	-0.21	-0.29,-0.13	4.41e-07	0.08	0.00,0.16	0.043	0.06	-0.02,0.14	0.162
**Cigarette smoking**	14399	1.25	1.18,1.33	1.46e-13	1.01	0.95,1.08	0.725	1.13	1.06,1.21	1.09e-04
**Alcohol consumption**	14399	0.96	0.92,0.99	0.016	1.04	1.00,1.08	0.033	1.02	0.99,1.06	0.214
**Binge drinking**	14399	0.97	0.92,1.02	0.203	1.01	0.96,1.06	0.763	1.05	1.00,1.10	0.038
**Coffee consumption**	14399	0.98	0.95,1.01	0.243	1.04	1.00,1.07	0.051	1.09	1.05,1.13	1.67e-06
**Binge coffee drinking**	14399	1.18	1.03,1.35	0.019	1.03	0.89,1.19	0.724	1.12	0.96,1.30	0.152
**No supplements taken**	14399	1.09	1.04,1.14	1.98e-04	1.01	0.97,1.06	0.543	0.93	0.89,0.97	0.001
**Folate supplement before pregnancy**	14399	0.96	0.93,0.99	0.017	1.02	0.99,1.06	0.182	1.01	0.97,1.04	0.637
**Folate supplement during pregnancy**	14399	0.93	0.88,0.97	0.001	0.97	0.93,1.02	0.235	1.04	0.99,1.09	0.099
**Metabolic conditions**										
**BMI before pregnancy***	14075	0.25	0.18,0.33	2.01e-11	0.06	-0.02,0.14	0.126	-0.18	-0.25,-0.10	4.67e-06
**Weight gain***	12205	0.18	0.07,0.29	0.001	-0.02	-0.13,0.09	0.707	0.16	0.04,0.27	0.007
**Type 2 diabetes (incl. gestational diabetes)**	14399	0.94	0.79,1.11	0.445	1.09	0.92,1.29	0.313	0.96	0.83,1.11	0.579
**High blood pressure (incl. preeclampsia)**	14399	1.00	0.95,1.05	0.989	0.98	0.93,1.03	0.386	1.01	0.96,1.06	0.815
**Infectious and autoimmune diseases**										
**Hyper-/Hypothyroidism**	14399	1.09	0.96,1.24	0.167	1.00	0.85,1.18	0.974	1.00	0.86,1.15	0.945
**Upper respiratory tract infections**	14399	1.03	0.98,1.09	0.237	1.00	0.95,1.06	0.924	1.00	0.95,1.05	0.964
**Lower respiratory tract infections**	14399	1.03	0.94,1.14	0.525	1.04	0.94,1.16	0.440	1.02	0.92,1.13	0.720
**Fever**	14399	1.04	0.99,1.09	0.114	1.00	0.95,1.05	0.907	1.02	0.98,1.07	0.357
**Asthma**	14399	1.19	1.09,1.30	9.38e-05	1.08	0.99,1.17	0.095	0.96	0.88,1.04	0.302
**Psoriasis**	14399	0.99	0.88,1.12	0.885	1.03	0.91,1.17	0.625	0.87	0.76,0.99	0.032
**Type 1 diabetes**	14399	0.94	0.70,1.25	0.651	0.95	0.73,1.25	0.727	1.03	0.80,1.33	0.801
**Other autoimmune disease**	14399	0.93	0.80,1.08	0.360	1.03	0.88,1.20	0.707	0.95	0.82,1.10	0.490
**Other physical health conditions**										
**Vaginal bleeding**	14399	1.29	0.90,1.85	0.162	0.89	0.63,1.24	0.482	1.15	0.86,1.54	0.338
**B12 insufficiency**	14399	1.02	0.89,1.17	0.745	0.92	0.82,1.03	0.158	1.03	0.92,1.16	0.577
**Anaemia/low hemoglobin**	14399	0.93	0.84,1.03	0.149	0.96	0.86,1.08	0.538	1.07	0.95,1.20	0.281
**Indication for medicine use**										
**Depression/anxiety**	14399	1.15	1.08,1.22	7.63e-06	1.14	1.07,1.21	2.59e-05	1.12	1.05,1.19	2.10e-04
**Med Depression**	14399	1.23	1.02,1.48	0.033	1.00	0.84,1.18	0.957	1.42	1.21,1.68	3.00e-05
**Med Depression or anxiety**	14399	1.15	0.97,1.36	0.097	1.00	0.86,1.16	0.993	1.39	1.19,1.62	2.82e-05
**Pain**	14399	1.05	1.01,1.09	0.008	1.02	0.98,1.06	0.325	1.02	0.98,1.06	0.236
**Migraine**	14399	1.13	1.05,1.22	9.65e-04	1.13	1.05,1.22	0.002	0.98	0.91,1.06	0.646
**Epilepsy**	14399	0.77	0.56,1.06	0.113	0.86	0.66,1.13	0.279	0.95	0.72,1.25	0.699
**Headache**	14399	1.05	1.00,1.09	0.029	0.98	0.94,1.02	0.417	1.01	0.97,1.06	0.511
**Med Pain**	14399	1.09	1.02,1.17	0.018	1.05	0.98,1.14	0.180	1.03	0.96,1.11	0.346
**Med Fever**	14399	0.98	0.87,1.11	0.758	1.01	0.88,1.16	0.870	0.93	0.82,1.06	0.305
**Med Pain or fever**	14399	1.07	1.00,1.14	0.051	1.04	0.97,1.12	0.242	1.01	0.95,1.08	0.733

* Effect estimates for maternal age at birth, BMI and weight gain during pregnancy are shown as beta per 1 SD increase in PGS. Measures occurred during pregnancy unless otherwise specified. Multiple testing corrected p-value p<0.002.

© 2022 Havdahl A et al. *JAMA Psychiatry.*

eAppendix 6. Multiple Imputation (MI)

Since the pseudo r-squared of the IPW model was relatively low, we also performed chained equations multiple imputation using the *mi impute chained* command in STATA 16. We imputed all incomplete variables (n=14, see Table S8) to the total number of participants who responded at the first questionnaire (N = 86,076). One MI was conducted including all outcomes, exposures and auxiliary variables simultaneously (75 variables with n=100 iterations). As auxiliary variables, we included all variables identified to be predictive of missingness in the IPW model (n=32, Table S6) as well as all pregnancy related factors from the main analysis model. A summary of missing data which were imputed can be found in Table S8, and we assumed this data to be missing not at random. All variables were imputed in the same model using the *regress* command for continuous variables and *logit* command for binary variables. Truncated imputation was performed for gestational length (minimum 20 weeks), birthweight (minimum 500g), maternal age (minimum 16 years at childbirth), maternal ADHD symptoms (between 0 and 24), maternal BMI before pregnancy (minimum 12.5 kg/m^2) AGPAR scores (between 0 and 10). Results of the MI analysis are compared with the complete case results in Supplementary Figure S4.

eTable 8. Summary of variables with missing data before and after imputation.

	**Missingness [%]**	**Before imputation**		**After imputation**	
		**mean**	**SD**	**mean**	**SD**
PGS_{ADHD}	83.16	0.010	1.01	0.010	1.01
PGS_{Autism}	83.16	0.016	1.01	0.016	1.01
PGS_{SCZ}	83.16	0.016	1.00	0.016	1.00
Birthweight [g]	0.23	3550.08	615.75	3550.04	615.86
Gestational age [weeks]	0.43	39.34	2.28	39.34	2.28
Maternal age [years]	0.06	30.00	4.66	30.00	4.66
Maternal BMI [kg/m2]	2.76	24.02	4.29	24.02	4.29
APGAR score 1min	0.45	8.61	1.31	8.61	1.31
APGAR score 5min	0.44	9.36	1.01	9.36	1.01
Maternal ADHD symptoms	45.43	6.82	3.38	6.82	3.38
Weight gain [kg]	20.53	15.08	5.90	15.08	5.90
		$\mathbf{N_{case}}$	$\mathbf{\%_{case}}$	$\mathbf{N_{case}}$	$\mathbf{\%_{case}}$
Ever smoking	3.76	41,652	50.28	43,621	50.68
Ever consumed alcohol	4.51	78,660	95.70	82,291	95.60
Lifetime depression	3.55	20,306	24.46	21,094	24.51

© 2022 Havdahl A et al. *JAMA Psychiatry.*

eTable 9. The association between PGS for neurodevelopmental conditions and pregnancy-related factors after multiple imputation (N = 86,076)

	PGS ADHD			PGS Autism			PGS Schizophrenia		
	OR	95% CI	p	OR	95% CI	p	OR	95% CI	p
**Behavior and lifestyle**									
Maternal age	-0.23	-0.31,-0.14	2.18e-07	0.07	-0.01,0.15	0.082	0.04	-0.03,0.11	0.279
Cigarette smoking	1.27	1.20,1.35	2.90e-12	1.00	0.94,1.05	0.885	1.13	1.07,1.20	1.89e-05
Alcohol consumption	0.96	0.93,1.00	0.029	1.04	1.01,1.08	0.021	1.03	1.00,1.07	0.059
Binge drinking	0.99	0.94,1.03	0.538	1.01	0.96,1.06	0.762	1.06	1.00,1.11	0.033
Coffee consumption	0.99	0.95,1.02	0.463	1.04	1.00,1.07	0.025	1.09	1.05,1.12	6.45e-07
Binge coffee drinking	1.20	1.03,1.40	0.017	1.01	0.88,1.15	0.903	1.16	1.00,1.34	0.051
No supplements taken	1.09	1.04,1.13	8.30e-05	1.01	0.97,1.05	0.551	0.94	0.90,0.98	0.004
Folate supplement before pregnancy	0.96	0.93,0.99	0.017	1.02	0.98,1.05	0.341	1.00	0.97,1.03	0.916
**Metabolic conditions**									
BMI before pregnancy	0.26	0.19,0.33	2.43e-12	0.07	0.01,0.13	0.034	-0.18	-0.26,-0.11	3.31e-06
Weight gain	0.21	0.09,0.33	4.73e-04	0.00	-0.10,0.10	0.986	0.18	0.08,0.28	7.17e-04
High blood pressure (incl. preeclampsia)	1.00	0.96,1.04	0.887	0.98	0.94,1.03	0.425	1.00	0.95,1.05	0.960
Type 2 diabetes (incl. gestational diabetes)	0.92	0.79,1.07	0.270	1.05	0.89,1.22	0.569	0.96	0.83,1.11	0.546
Hyper-/Hypothyroidism	1.10	0.98,1.24	0.104	0.99	0.86,1.13	0.845	1.01	0.88,1.17	0.839
**Infections and immunological conditions**									
Upper respiratory tract infections	1.04	0.99,1.08	0.117	1.01	0.96,1.06	0.723	1.00	0.95,1.05	0.906
Lower respiratory tract infections	1.04	0.94,1.15	0.442	1.05	0.94,1.17	0.420	1.00	0.91,1.11	0.963
Urinary tract infection	1.07	1.01,1.12	0.021	1.08	1.02,1.15	0.010	1.05	1.00,1.10	0.051
Fever	1.02	0.98,1.07	0.278	0.99	0.95,1.04	0.726	1.03	0.98,1.07	0.239
Asthma	1.17	1.07,1.27	4.79e-04	1.07	0.98,1.17	0.124	0.96	0.88,1.05	0.348
Psoriasis	1.00	0.88,1.13	0.956	1.01	0.89,1.15	0.850	0.90	0.79,1.02	0.098
Type 1 diabetes	0.93	0.74,1.18	0.543	0.97	0.74,1.27	0.822	0.97	0.75,1.26	0.822
Other autoimmune disease	0.93	0.80,1.09	0.376	1.05	0.91,1.21	0.511	0.94	0.83,1.07	0.372
**Other physical health conditions**									
Vaginal bleeding	1.18	0.81,1.73	0.386	0.86	0.59,1.24	0.411	1.16	0.81,1.67	0.419
B12 insufficiency	1.03	0.92,1.16	0.630	0.92	0.80,1.05	0.211	1.05	0.94,1.19	0.383
Anaemia/low hemoglobin	0.92	0.84,1.02	0.099	0.99	0.88,1.11	0.797	1.05	0.95,1.15	0.371
**Indication for medicine use**									
Depression/anxiety	1.16	1.09,1.24	6.73e-06	1.12	1.06,1.19	1.76e-04	1.14	1.07,1.20	1.20e-05
Med Depression	1.19	1.00,1.41	0.054	1.03	0.87,1.22	0.726	1.44	1.23,1.69	1.04e-05
Med Depression or anxiety	1.12	0.95,1.31	0.184	1.03	0.89,1.21	0.660	1.39	1.19,1.61	3.50e-05
Pain	1.06	1.02,1.10	0.003	1.01	0.97,1.05	0.677	1.02	0.98,1.06	0.270
Headache	1.03	0.99,1.08	0.112	0.98	0.94,1.02	0.368	1.01	0.98,1.05	0.501
Migraine	1.12	1.05,1.19	3.02e-04	1.10	1.02,1.18	0.013	0.99	0.92,1.07	0.858
Epilepsy	0.82	0.60,1.13	0.229	0.83	0.62,1.12	0.215	0.95	0.71,1.28	0.750
Med Pain or fever	1.05	0.99,1.12	0.087	1.02	0.96,1.08	0.446	1.02	0.96,1.10	0.476
Med Pain	1.07	1.00,1.14	0.051	1.04	0.97,1.11	0.314	1.05	0.97,1.13	0.205
Med Fever	1.00	0.88,1.13	0.988	1.01	0.88,1.16	0.885	0.95	0.84,1.07	0.376

Note. All measures occurred during pregnancy unless otherwise specified. * Effect estimates for maternal age at birth, BMI and weight gain during pregnancy are shown as beta per 1 SD increase in PGS. Multiple testing corrected p-value p<0.002.

© 2022 Havdahl A et al. *JAMA Psychiatry.*

eAppendix 7. ADHD symptom measures in adulthood in MoBa

Parental symptoms of ADHD were assessed as the sum of symptoms on the 6-item DSM-IV Adult ADHD Self-Report Scale (ASRS) at study recruitment (fathers) and at the year 3 follow-up (mothers). Of the 6 items, 4 capture inattention (e.g. "How often do you have problems remembering appointments or obligations?") and 2 capture hyperactivity (e.g. "How often do you feel overly active and compelled to do things, like you were driven by a motor?"). Responses were given on a 5-point likert scale (0 = never, 1 = rarely, 2=sometimes, 3 = often, 4 = very often), so summed scores ranged from 0-24. A cut-off of 14 can be used to suggest possible ADHD[34].

Statistical analysis

We regressed the standardised PGS (constructed at p-value threshold $p<0.05$) against ADHD symptoms in adulthood. The regression was run separately for males and females, and was adjusted for 10 principal components of population structure.

© 2022 Havdahl A et al. *JAMA Psychiatry.*

eAppendix 8. Paternal PGS sample overview and results

eTable 10. Sample overview of father pregnancy-related factors in MoBa (full cohort and genotyped cohort compared)

	**Full Sample**		**Genotyped Sample**		
	**N(No)**	**N(Yes)(%)**	**N(No)**	**N(Yes)(%)**	**P-value**
**Paternal behavior and lifestyle**					
Ever smoked	30961	35005 (53.1)	6781	7350 (52)	0.024
Cigarette smoking	23204	16224 (41.1)	4901	3347 (40.6)	0.346
Ever drank alcohol	1633	64419 (97.5)	296	13866 (97.9)	0.008
Binge drinking before pregnancy	2555	23174 (90.1)	415	4216 (91)	0.044
Coffee consumption	44867	21669 (32.6)	10383	3872 (27.2)	2.55e-36
Binge coffee drinking	60492	6044 (9.1)	13192	1063 (7.5)	5.42e-10
No supplements taken	10704	15685 (59.4)	1919	2800 (59.3)	0.907
Diabetes	65937	599 (0.9)	14130	125 (0.9)	0.826
**Paternal illness and physical health conditions**					
High blood pressure	64633	1903 (2.9)	13844	411 (2.9)	0.903
Asthma	60501	6035 (9.1)	12939	1316 (9.2)	0.553
Psoriasis	63752	2784 (4.2)	13655	600 (4.2)	0.911
Other autoimmune disease	63838	2698 (4.1)	13746	509 (3.6)	0.008
Pain	48692	17844 (26.8)	10552	3703 (26)	0.040
Headache	61562	4974 (7.5)	13192	1063 (7.5)	0.953
Migraine	61937	4599 (6.9)	13272	983 (6.9)	0.959
Lifetime depression	58445	6927 (10.6)	12625	1401 (10)	0.034

Note. All measures occurred during the partners pregnancy, unless otherwise specified. Multiple testing corrected p-value p<0.002.

© 2022 Havdahl A et al. *JAMA Psychiatry.*

eTable 11. Association of paternal polygenic risk scores (PGS) for attention deficit hyperactivity disorder (ADHD), autism and schizophrenia (SCZ) with pregnancy related factors measured in fathers.

		PGS ADHD			PGS Autism			PGS Schizophrenia		
	N	OR	95% CI	p	OR	95% CI	p	OR	95% CI	p
**Paternal behavior and lifestyle**										
Paternal age	14887	-0.03	-0.11,0.06	0.522	0.10	0.02,0.18	0.016	0.15	0.07,0.23	3.02e-04
Ever smoked	14131	1.13	1.09,1.17	4.78e-13	1.04	1.01,1.08	0.011	1.08	1.05,1.12	2.15e-06
Cigarette smoking	8248	1.06	1.01,1.10	0.014	1.00	0.95,1.04	0.916	1.02	0.98,1.07	0.354
Ever drank alcohol	14162	1.06	0.94,1.19	0.334	1.04	0.93,1.17	0.520	1.25	1.11,1.40	1.55e-04
Binge drinking before pregnancy	4631	1.01	0.92,1.12	0.779	1.11	1.00,1.23	0.043	1.01	0.92,1.12	0.778
Binge drinking	4541	1.06	0.98,1.14	0.177	1.01	0.94,1.10	0.742	1.01	0.93,1.09	0.899
Coffee consumption	14255	0.98	0.95,1.02	0.329	1.01	0.97,1.05	0.587	0.98	0.95,1.02	0.304
Binge coffee drinking	14255	1.02	0.95,1.08	0.632	1.02	0.96,1.09	0.459	1.01	0.95,1.08	0.724
No supplements taken	4719	1.00	0.95,1.06	0.926	1.02	0.96,1.08	0.536	0.91	0.86,0.97	0.002
**Paternal illness and conditions**										
BMI	4762	0.12	0.02,0.22	0.018	-0.08	-0.18,0.02	0.117	-0.05	-0.15,0.05	0.358
Diabetes	14255	0.97	0.81,1.15	0.713	1.02	0.86,1.22	0.800	1.00	0.84,1.19	0.991
High blood pressure	14255	1.08	0.98,1.19	0.108	0.98	0.89,1.08	0.642	1.03	0.94,1.14	0.511
Asthma	14255	1.07	1.01,1.13	0.027	1.03	0.98,1.09	0.266	1.04	0.98,1.10	0.192
Psoriasis	14255	1.06	0.97,1.15	0.181	1.02	0.94,1.11	0.670	1.03	0.95,1.12	0.471
Other autoimmune disease	14255	1.05	0.96,1.15	0.254	1.04	0.95,1.13	0.444	0.98	0.90,1.07	0.629
Pain	14255	1.09	1.05,1.13	6.07e-06	1.00	0.97,1.04	0.916	1.00	0.96,1.03	0.820
Headache	14255	1.11	1.04,1.18	0.001	1.03	0.96,1.09	0.422	1.01	0.95,1.07	0.804
Migraine	14255	1.06	1.00,1.13	0.062	0.99	0.93,1.05	0.699	1.02	0.95,1.08	0.644
Lifetime depression	14026	1.07	1.02,1.13	0.012	1.07	1.01,1.13	0.019	1.18	1.12,1.25	2.67e-09

Note. All measures occurred during the partners pregnancy, unless otherwise specified. * Effect estimates for paternal age at birth and BMI are shown as beta per 1 SD increase in PGS. Multiple testing corrected p-value p<0.002.

© 2022 Havdahl A et al. *JAMA Psychiatry.*

eAppendix 9. PGS associations at different p-value thresholds

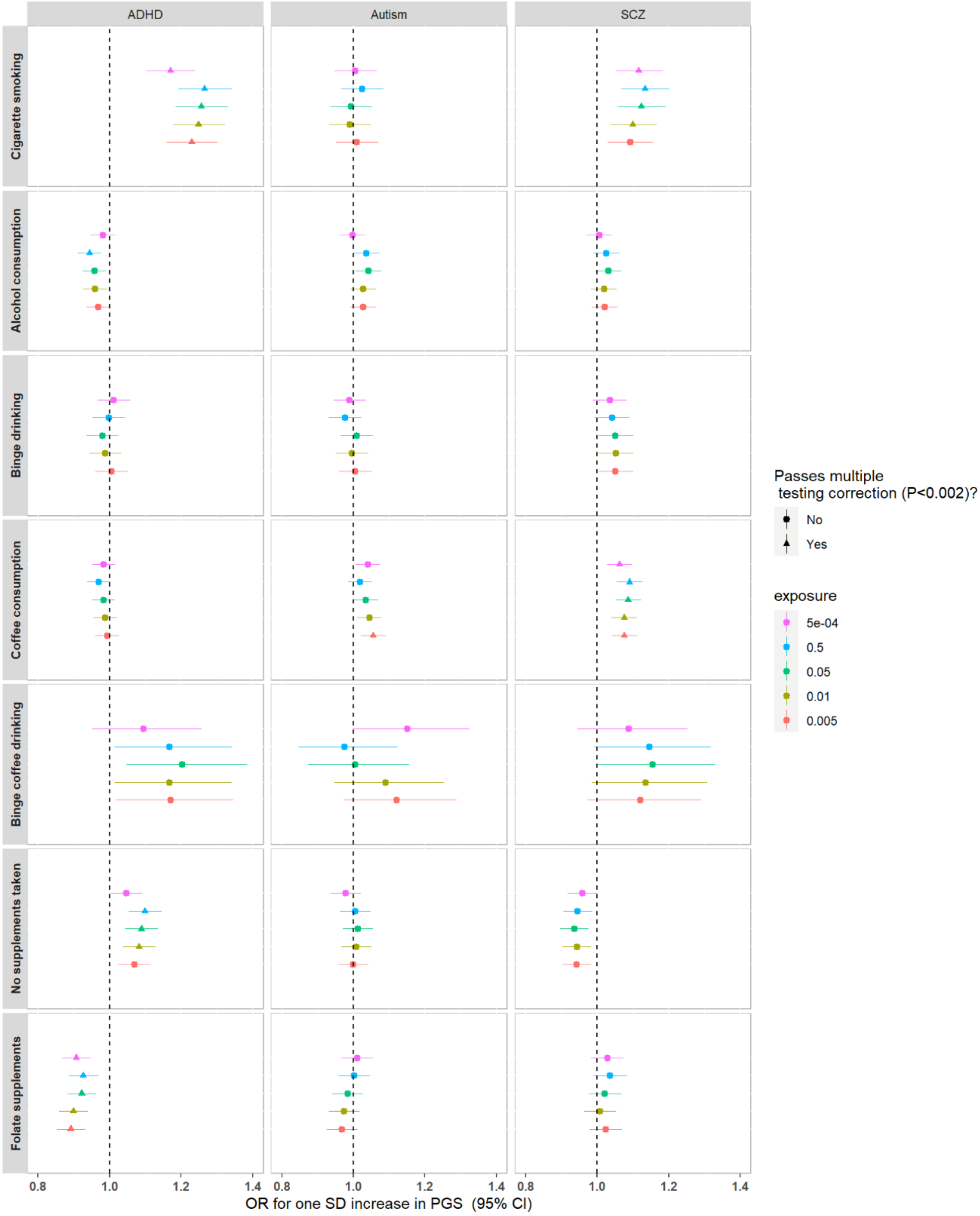

eFigure 3a. Association of behaviour and lifestyle measures with polygenic risk scores for ADHD, autism and schizophrenia across different p-value thresholds.

© 2022 Havdahl A et al. *JAMA Psychiatry.*

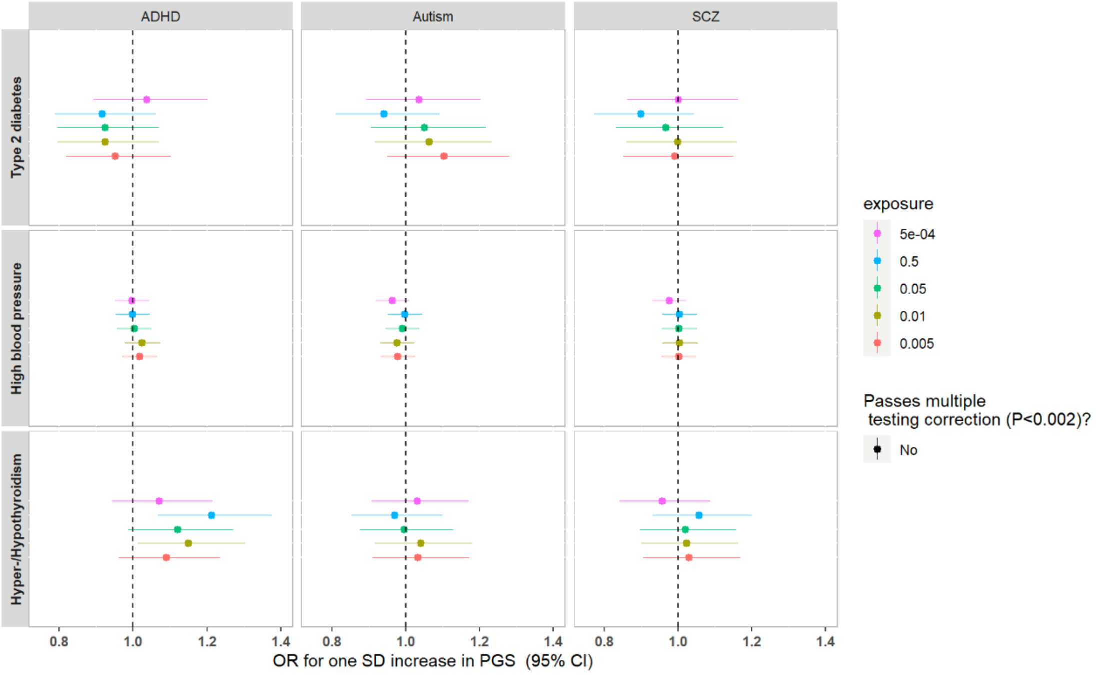

eFigure 3b. Association of metabolic conditions with polygenic risk scores for ADHD, autism and schizophrenia across different p-value thresholds.

© 2022 Havdahl A et al. *JAMA Psychiatry.*

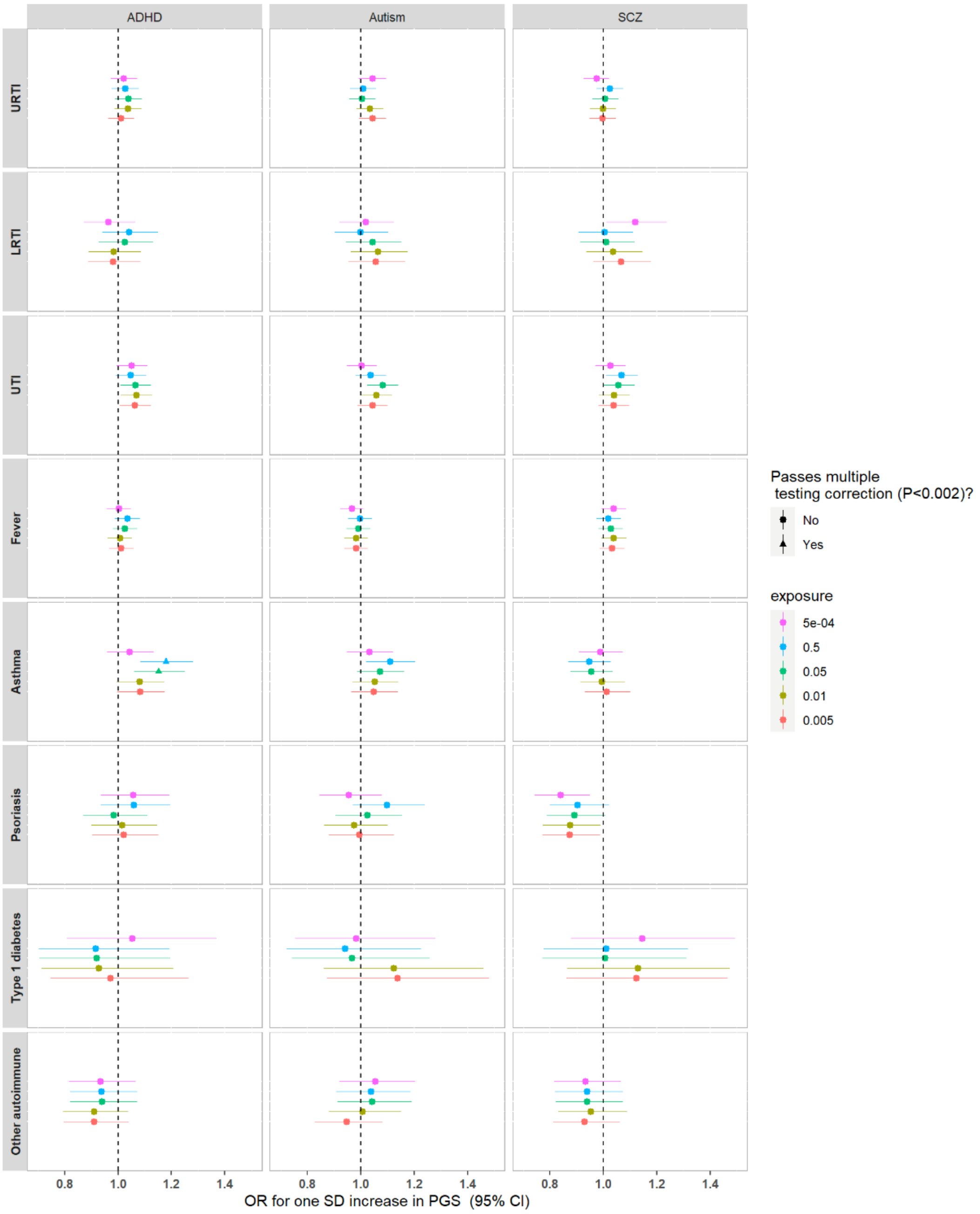

eFigure 3c. Association of autoimmune and infectious diseases with polygenic risk scores for ADHD, autism and schizophrenia across different p-value thresholds.

© 2022 Havdahl A et al. *JAMA Psychiatry*.

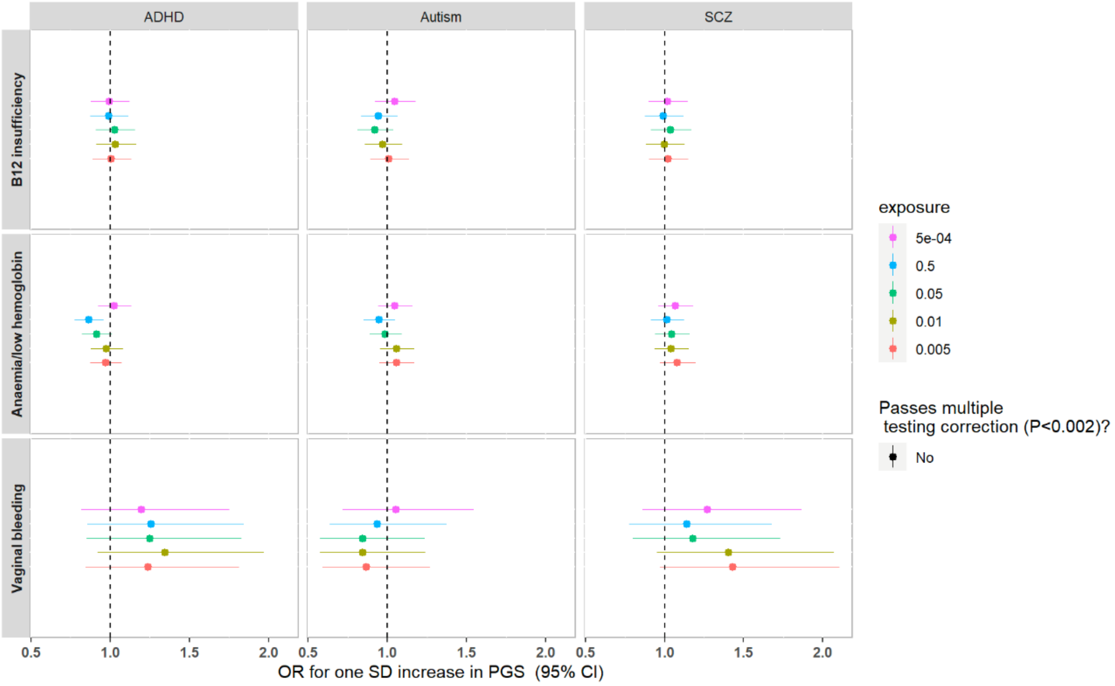

eFigure 3d. Association of other physical health conditions with polygenic risk scores for ADHD, autism and schizophrenia across different p-value thresholds.

© 2022 Havdahl A et al. *JAMA Psychiatry.*

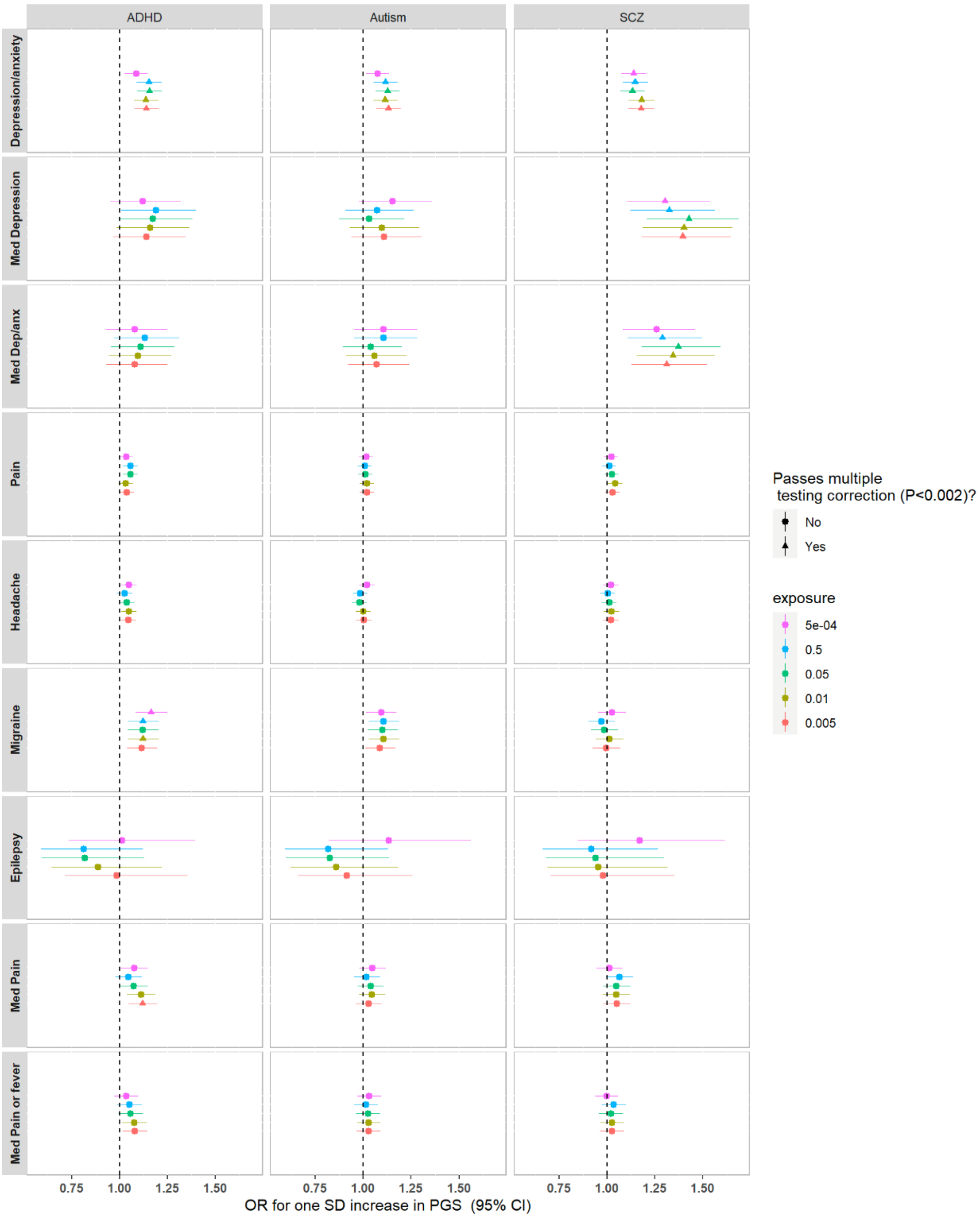

eFigure 3e. Association of medication use (or health problems suggestive of possible medication use) with polygenic risk scores for ADHD, autism and schizophrenia across different p-value thresholds.

© 2022 Havdahl A et al. *JAMA Psychiatry.*

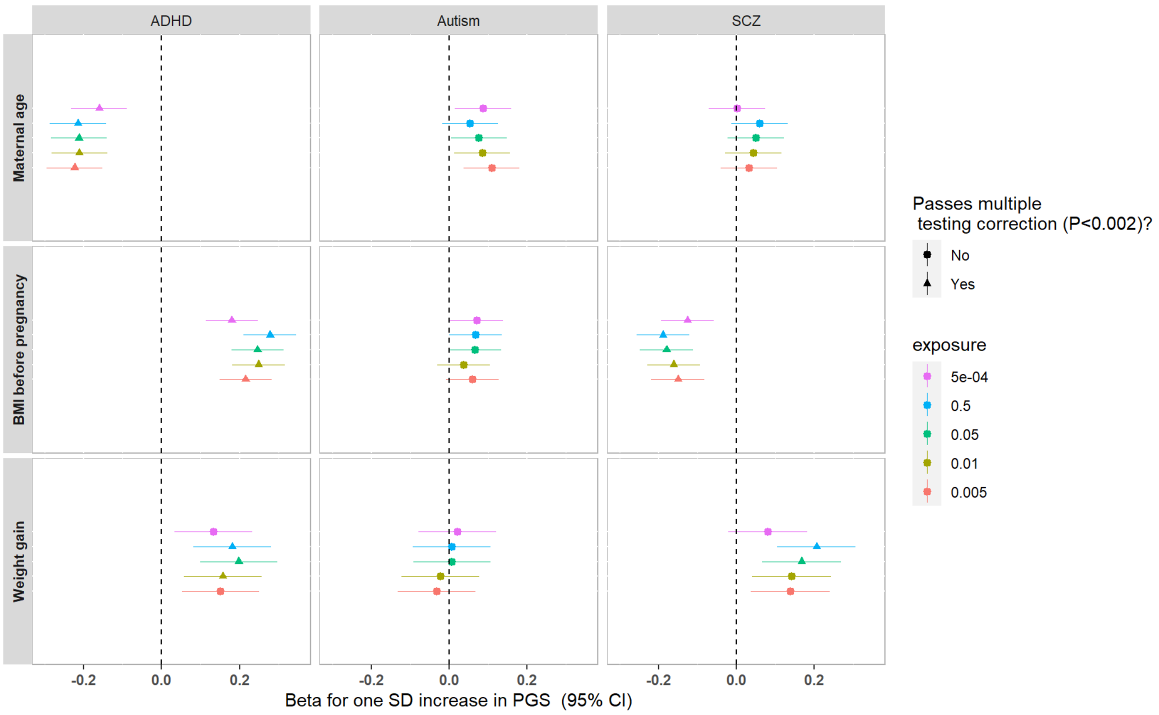

eFigure 3f. Association of continuous measures with polygenic risk scores for ADHD, autism and schizophrenia across different p-value thresholds.

© 2022 Havdahl A et al. *JAMA Psychiatry.*

eAppendix 10. Comparison of complete case, inverse probability weighted and multiple imputation results

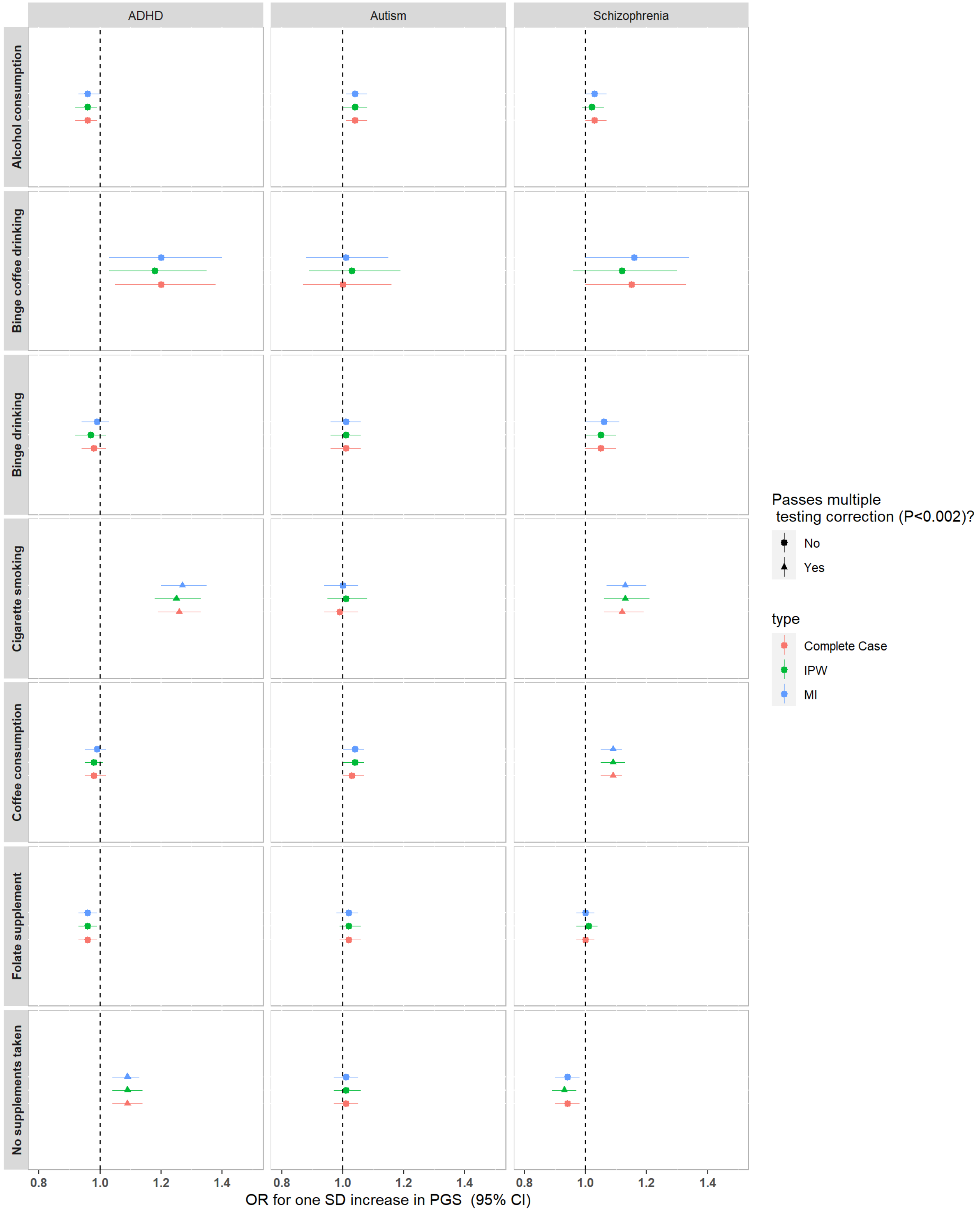

eFigure 4a. Associations between behaviour and lifestyle measures with polygenic risk scores for ADHD, autism and schizophrenia comparing results from complete case analysis, inverse probability weighting (IPW) and multiple imputation (MI).

© 2022 Havdahl A et al. *JAMA Psychiatry.*

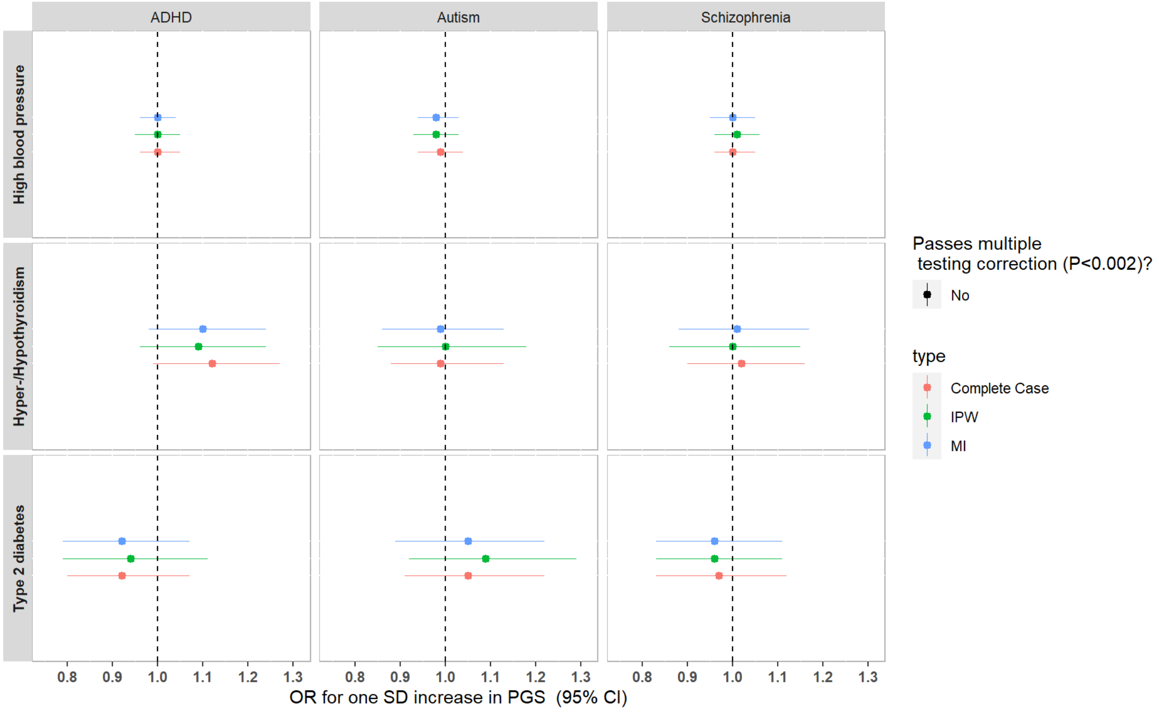

eFigure 4b. Associations between metabolic measures with polygenic risk scores for ADHD, autism and schizophrenia comparing results from complete case analysis, inverse probability weighting (IPW) and multiple imputation (MI).

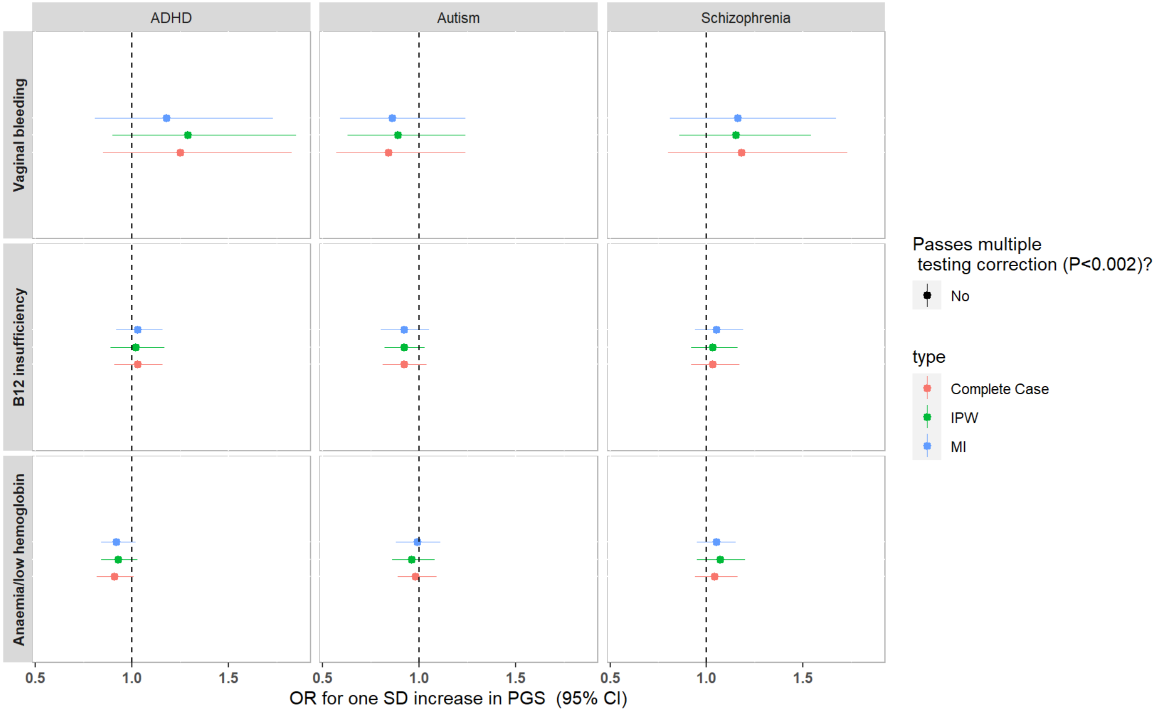

eFigure 4c. Associations between other physical health conditions with polygenic risk scores for ADHD, autism and schizophrenia comparing results from complete case analysis, inverse probability weighting (IPW) and multiple imputation (MI).

© 2022 Havdahl A et al. *JAMA Psychiatry.*

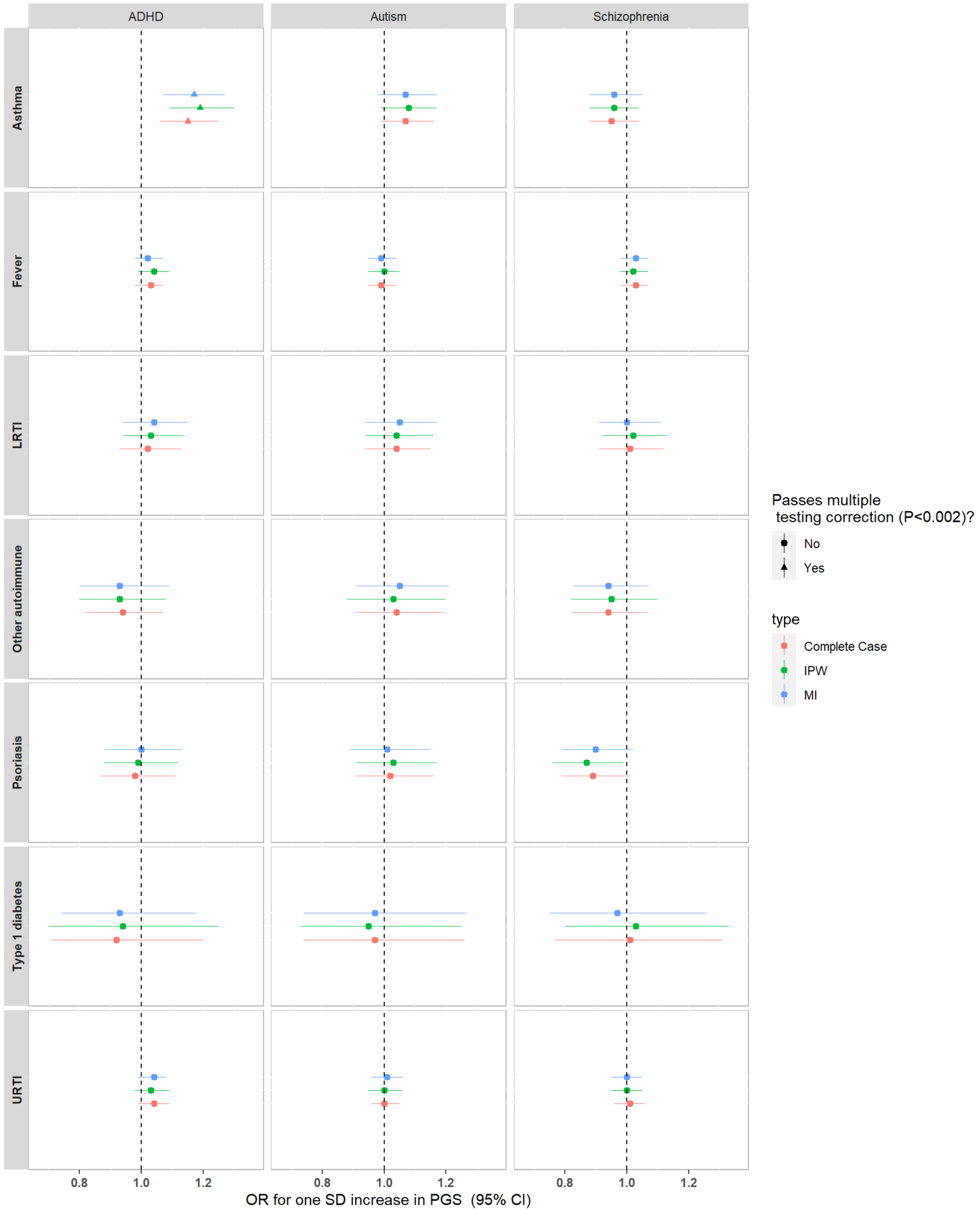

eFigure 4d. Associations between autoimmune and infectious diseases with polygenic risk scores for ADHD, autism and schizophrenia comparing results from complete case analysis, inverse probability weighting (IPW) and multiple imputation (MI).

© 2022 Havdahl A et al. *JAMA Psychiatry.*

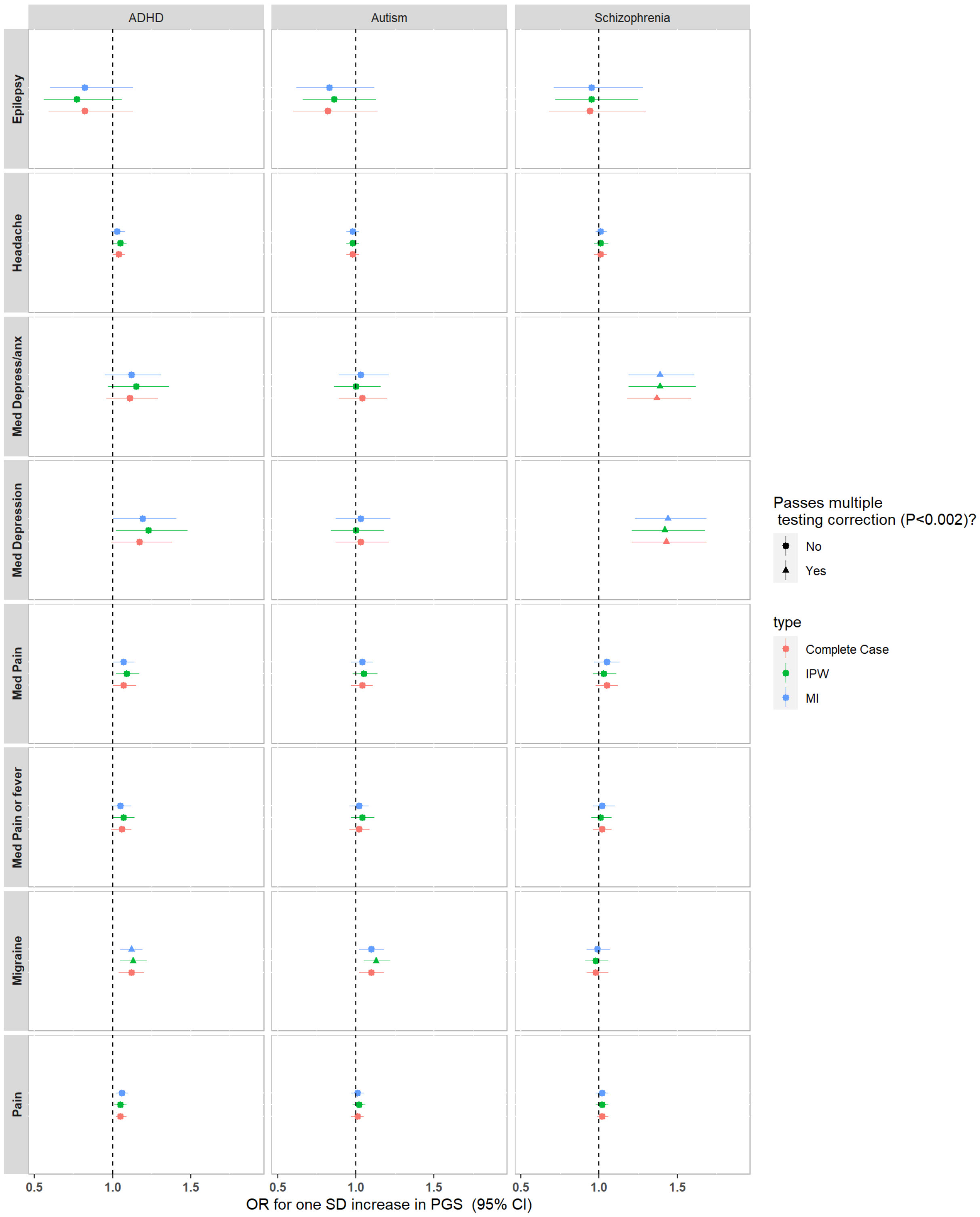

eFigure 4e. Associations between indications for medication use with polygenic risk scores for ADHD, autism and schizophrenia comparing results from complete case analysis, inverse probability weighting (IPW) and multiple imputation (MI).

© 2022 Havdahl A et al. *JAMA Psychiatry.*

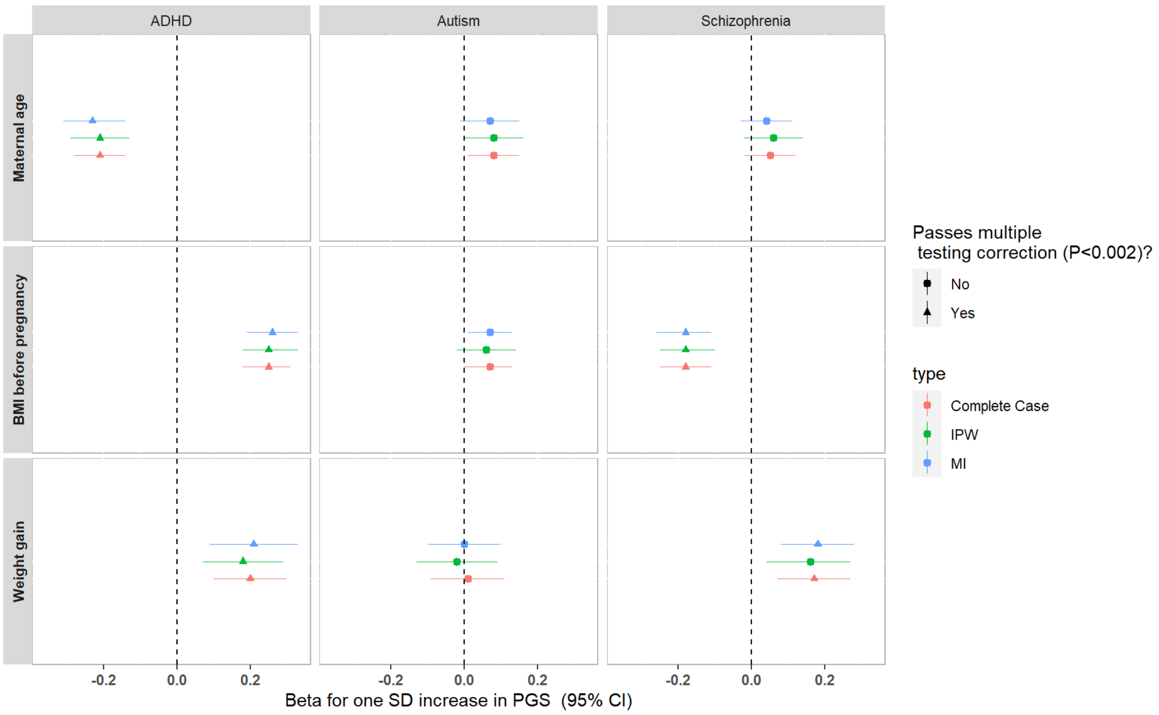

eFigure 4f. Associations between continuous traits with polygenic risk scores for ADHD, autism and schizophrenia comparing results from complete case analysis, inverse probability weighting (IPW) and multiple imputation (MI).

© 2022 Havdahl A et al. *JAMA Psychiatry.*

eReferences

1. Helgeland Ø, Vaudel M, Juliusson PB, et al. Genome-wide association study reveals dynamic role of genetic variation in infant and early childhood growth. *Nat Commun*. 2019;10(1):4448. doi:10.1038/s41467-019-12308-0

2. Chang Z, Lichtenstein P, D'Onofrio BM, et al. Maternal age at childbirth and risk for ADHD in offspring: a population-based cohort study. *Int J Epidemiol*. 2014;43(6):1815-1824. doi:10.1093/ije/dyu204

3. Gustavson K, Ystrom E, Stoltenberg C, et al. Smoking in Pregnancy and Child ADHD. *Pediatrics*. 2017;139(2). doi:10.1542/peds.2016-2509

4. He Y, Chen J, Zhu L-H, Hua L-L, Ke F-F. Maternal Smoking During Pregnancy and ADHD: Results From a Systematic Review and Meta-Analysis of Prospective Cohort Studies. *J Atten Disord*. Published online March 1, 2017:1087054717696766. doi:10.1177/1087054717696766

5. Sciberras E, Mulraney M, Silva D, Coghill D. Prenatal Risk Factors and the Etiology of ADHD-Review of Existing Evidence. *Curr Psychiatry Rep*. 2017;19(1):1. doi:10.1007/s11920-017-0753-2

6. Sayal K, Heron J, Draper E, et al. Prenatal exposure to binge pattern of alcohol consumption: mental health and learning outcomes at age 11. *Eur Child Adolesc Psychiatry*. 2014;23(10):891-899. doi:10.1007/s00787-014-0599-7

7. Linnet KM, Wisborg K, Secher NJ, et al. Coffee consumption during pregnancy and the risk of hyperkinetic disorder and ADHD: a prospective cohort study. *Acta Paediatr Oslo Nor 1992*. 2009;98(1):173-179. doi:10.1111/j.1651-2227.2008.00980.x

8. Desai A, Sequeira JM, Quadros EV. The metabolic basis for developmental disorders due to defective folate transport. *Biochimie*. 2016;126:31-42. doi:10.1016/j.biochi.2016.02.012

9. Meli G, Ottl B, Paladini A, Cataldi L. Prenatal and perinatal risk factors of schizophrenia. *J Matern-Fetal Neonatal Med Off J Eur Assoc Perinat Med Fed Asia Ocean Perinat Soc Int Soc Perinat Obstet*. 2012;25(12):2559-2563. doi:10.3109/14767058.2012.699118

10. Surén P, Roth C, Bresnahan M, et al. Association between maternal use of folic acid supplements and risk of autism spectrum disorders in children. *JAMA*. 2013;309(6):570-577. doi:10.1001/jama.2012.155925

11. Martins-Silva T, Vaz JDS, Hutz MH, et al. Assessing causality in the association between attention-deficit/hyperactivity disorder and obesity: a Mendelian randomization study. *Int J Obes 2005*. 2019;43(12):2500-2508. doi:10.1038/s41366-019-0346-8

12. Van Lieshout RJ, Taylor VH, Boyle MH. Pre-pregnancy and pregnancy obesity and neurodevelopmental outcomes in offspring: a systematic review. *Obes Rev*. 2011;12(5):e548-e559. doi:10.1111/j.1467-789X.2010.00850.x

13. Wang C, Geng H, Liu W, Zhang G. Prenatal, perinatal, and postnatal factors associated with autism: A meta-analysis. *Medicine (Baltimore)*. 2017;96(18):e6696. doi:10.1097/MD.0000000000006696

© 2022 Havdahl A et al. *JAMA Psychiatry.*

14. Dachew BA, Scott JG, Mamun A, Alati R. Pre-eclampsia and the risk of attention-deficit/hyperactivity disorder in offspring: Findings from the ALSPAC birth cohort study. *Psychiatry Res*. 2019;272:392-397. doi:10.1016/j.psychres.2018.12.123

15. Hoirisch-Clapauch S, Nardi AE. Autism spectrum disorders: let's talk about glucose? *Transl Psychiatry*. 2019;9(1):51. doi:10.1038/s41398-019-0370-4

16. Gardener H, Spiegelman D, Buka SL. Prenatal risk factors for autism: comprehensive meta-analysis. *Br J Psychiatry J Ment Sci*. 2009;195(1):7-14. doi:10.1192/bjp.bp.108.051672

17. Milberger S, Biederman J, Faraone SV, Guite J, Tsuang MT. Pregnancy, delivery and infancy complications and attention deficit hyperactivity disorder: issues of gene-environment interaction. *Biol Psychiatry*. 1997;41(1):65-75. doi:10.1016/0006-3223(95)00653-2

18. Wiegersma AM, Dalman C, Lee BK, Karlsson H, Gardner RM. Association of Prenatal Maternal Anemia With Neurodevelopmental Disorders. *JAMA Psychiatry*. Published online September 18, 2019:1-12. doi:10.1001/jamapsychiatry.2019.2309

19. Hornig M, Bresnahan MA, Che X, et al. Prenatal fever and autism risk. *Mol Psychiatry*. 2018;23(3):759-766. doi:10.1038/mp.2017.119

20. Instanes JT, Halmøy A, Engeland A, Haavik J, Furu K, Klungsøyr K. Attention-Deficit/Hyperactivity Disorder in Offspring of Mothers With Inflammatory and Immune System Diseases. *Biol Psychiatry*. 2017;81(5):452-459. doi:10.1016/j.biopsych.2015.11.024

21. Jiang H-Y, Xu L-L, Shao L, et al. Maternal infection during pregnancy and risk of autism spectrum disorders: A systematic review and meta-analysis. *Brain Behav Immun*. 2016;58:165-172. doi:10.1016/j.bbi.2016.06.005

22. Werenberg Dreier J, Nybo Andersen A-M, Hvolby A, Garne E, Kragh Andersen P, Berg-Beckhoff G. Fever and infections in pregnancy and risk of attention deficit/hyperactivity disorder in the offspring. *J Child Psychol Psychiatry*. 2016;57(4):540-548. doi:10.1111/jcpp.12480

23. Dror DK, Allen LH. Interventions with vitamins B6, B12 and C in pregnancy. *Paediatr Perinat Epidemiol*. 2012;26 Suppl 1:55-74. doi:10.1111/j.1365-3016.2012.01277.x

24. Chen M-H, Su T-P, Chen Y-S, et al. Comorbidity of Allergic and Autoimmune Diseases Among Patients With ADHD: A Nationwide Population-Based Study. *J Atten Disord*. 2017;21(3):219-227. doi:10.1177/1087054712474686

25. Chen S-W, Zhong X-S, Jiang L-N, et al. Maternal autoimmune diseases and the risk of autism spectrum disorders in offspring: A systematic review and meta-analysis. *Behav Brain Res*. 2016;296:61-69. doi:10.1016/j.bbr.2015.08.035

26. Rom AL, Wu CS, Olsen J, Jawaheer D, Hetland ML, Mørch LS. Parental Rheumatoid Arthritis and Autism Spectrum Disorders in Offspring: A Danish Nationwide Cohort Study. *J Am Acad Child Adolesc Psychiatry*. 2018;57(1):28-32.e1. doi:10.1016/j.jaac.2017.10.002

27. Wu S, Ding Y, Wu F, et al. Family history of autoimmune diseases is associated with an increased risk of autism in children: A systematic review and meta-analysis. *Neurosci Biobehav Rev*. 2015;55:322-332. doi:10.1016/j.neubiorev.2015.05.004

© 2022 Havdahl A et al. *JAMA Psychiatry.*

28. Clements CC, Castro VM, Blumenthal SR, et al. Prenatal antidepressant exposure is associated with risk for attention-deficit hyperactivity disorder but not autism spectrum disorder in a large health system. *Mol Psychiatry*. 2015;20(6):727-734. doi:10.1038/mp.2014.90

29. Bauer AZ, Kriebel D, Herbert MR, Bornehag C-G, Swan SH. Prenatal paracetamol exposure and child neurodevelopment: A review. *Horm Behav*. 2018;101:125-147. doi:10.1016/j.yhbeh.2018.01.003

30. Stergiakouli E, Thapar A, Davey Smith G. Association of Acetaminophen Use During Pregnancy With Behavioral Problems in Childhood: Evidence Against Confounding. *JAMA Pediatr*. 2016;170(10):964-970. doi:10.1001/jamapediatrics.2016.1775

31. Brandlistuen RE, Ystrom E, Nulman I, Koren G, Nordeng H. Prenatal paracetamol exposure and child neurodevelopment: a sibling-controlled cohort study. *Int J Epidemiol*. 2013;42(6):1702-1713. doi:10.1093/ije/dyt183

32. Liew Z, Ritz B, Rebordosa C, Lee P-C, Olsen J. Acetaminophen use during pregnancy, behavioral problems, and hyperkinetic disorders. *JAMA Pediatr*. 2014;168(4):313-320. doi:10.1001/jamapediatrics.2013.4914

33. Gustavson K, Ystrom E, Ask H, et al. Acetaminophen use during pregnancy and offspring attention deficit hyperactivity disorder – a longitudinal sibling control study. *JCPP Adv*. 2021;1(2):e12020. doi:10.1002/jcv2.12020

34. Kessler RC, Adler L, Ames M, et al. The World Health Organization Adult ADHD Self-Report Scale (ASRS): a short screening scale for use in the general population. *Psychol Med*. 2005;35(2):245-256. doi:10.1017/s0033291704002892

© 2022 Havdahl A et al. *JAMA Psychiatry.*
